# Systematic Review: Adverse Events of Fecal Microbiota Transplantation

**DOI:** 10.1371/journal.pone.0161174

**Published:** 2016-08-16

**Authors:** Sinan Wang, Mengque Xu, Weiqiang Wang, Xiaocang Cao, Meiyu Piao, Samiullah Khan, Fang Yan, Hailong Cao, Bangmao Wang

**Affiliations:** 1 Department of Gastroenterology and Hepatology, General Hospital, Tianjin Medical University, Tianjin, China; 2 Division of Gastroenterology, Hepatology and Nutrition, Department of Pediatrics, Vanderbilt University Medical Center, Nashville, TN 37232, United States of America; Fox Chase Cancer Center, UNITED STATES

## Abstract

**Background:**

Fecal microbiota transplantation (FMT) is a microbiota-based therapy that shows therapeutic potential in recurrent or refractory *Clostridium difficile* infections and other intestinal or extra-intestinal disorders. Nonetheless, adverse events (AEs) remain a major challenge in the application of FMT.

**Aim:**

To review the AEs of FMT and to address the concerns of safety during the procedure.

**Methods:**

Publications were retrieved in the databases of Medline, Embase and Cochrane Library. AEs were classified according to their causality with FMT or their severity.

**Results:**

A total of 7562 original articles about FMT were identified in this study, 50 of them fulfilled the inclusion criteria. Totally 78 kinds of AEs were revealed enrolled in these 50 selected publications. The total incidence rate of AEs was 28.5%. Among the 42 publications, 5 kinds were definitely and 38 kinds were probably related to FMT. The commonest FMT-attributable AE was abdominal discomfort, which was reported in 19 publications. For upper gastrointestinal routes of FMT, 43.6% (89/204) patients were compromised by FMT-attributable AE, while the incidence dropped to 17.7% (76/430) for lower gastrointestinal routes. In contrast, the incidences of serious adverse events (SAEs) were 2.0% (4/196) and 6.1% (40/659) for upper and lower gastrointestinal routes, respectively. A total of 44 kinds of SAEs occurred in 9.2% patients, including death (3.5%, 38/1089), infection (2.5%, 27/1089), relapse of inflammatory bowel diseases (0.6%, 7/1089) and *Clostridium difficile* infection (0.9%, 10/1089).

**Conclusion:**

Consequently, both AEs and SAEs are not rare and should be carefully monitored throughout FMT. However, high quality randomized controlled trials are still needed for the more definite incidence of AEs of FMT.

## Introduction

The gut microbiota is one of the most complex systems in human body, which comprises about 10^14^ microbes, outnumbering human cells by 10-fold [1–3]. The majority of microbes have an extensive influence on human, including digestion, immunity, energy homeostasis, vitamin synthesis, etc. [4–7]. Alteration of the gut microbiota has been associated with both digestive and extra-digestive disorders [8–12]. Novel strategy for treatment of bacteria-associated diseases, via modulating the gut microbiota, is underway to establish its pivotal role.

Fecal microbiota transplantation (FMT), also known as fecal bacteriotherapy or intestinal microbiota transplantation, is defined as the perfusion of treated feces from a healthy donor via the upper or lower gastrointestinal route [13]. About 1700 years ago, Ge Hong, a well-known traditional Chinese medicine doctor, firstly described the use of human fecal suspension by mouth for patients with food poisoning or severe diarrhea [14]. In 1958, Eiseman *et al* applied FMT to treat antibiotic-associated diarrhea [15]. Since Schwan *et al* reported the first FMT therapy for CDI in 1983 [16], the application of FMT in CDI has been practiced extensively [17–19]. The effective rate of FMT for recurrent or refractory CDI was over 90% [20, 21]. Although FMT is still regarded as an investigational agent and requires an investigational new drug (IND) application, the US FDA has already recommended FMT as an alternative therapy for recurrence of CDI after a pulsed vancomycin regimen [22]. Moreover, FMT shows remarkable therapeutic potential in diverse conditions [13, 23] including inflammatory bowel diseases (IBD) [24, 25], irritable bowel syndrome (IBS) [26–28], metabolic diseases [4, 29, 30], neuropsychiatric conditions [31], autoimmune diseases [32, 33], allergic disorders [34, 35], and chronic fatigue syndrome [36].

Although patients benefit from FMT, concerns about this emerging strategy remain to be addressed, including long-term outcomes of FMT and the AEs. So far, the exact roles of the gut microbiota in FMT are not yet fully understood. And the AEs that happen during or after FMT still perplex clinicians and fundamental researchers. Hence, we systematically reviewed the AEs of FMT in all related publications aiming to elucidate the causality between FMT and the AEs. Furthermore, the AEs of FMT were divided into different degrees according to the severity and SAEs were emphatically introduced to arouse attention in FMT.

## Methods

### Information Sources and Search Strategy

Electronic databases for literature search included the Medline, Embase, and Cochrane Library. The last search was run on July 2015. The complete string used for the electronic search is shown in Table 1. All the deriving terms were combined by the Boolean operator “OR” to assure the identification of studies regarding FMT.

**Table 1:** Complete String Used for the Electronic Search.

(fecal microbiota transplantation) OR (fecal transplantation) OR (feces transplantation) OR (stool transplantation) OR (microflora transplantation) OR (fecal flora transplantation) OR (fecal transplant) OR (fecal microbiota transplant) OR (feces transplant) OR (stool transplant) OR (microflora transplant) OR (fecal flora transplant) OR (fecal bacteriotherapy) OR (fecal microbiota bacteriotherapy) OR (feces bacteriotherapy) OR (stool bacteriotherapy) OR (microflora bacteriotherapy) OR (fecal flora bacteriotherapy) OR (fecal suspension) OR (fecal microbiota suspension) OR (feces suspension) OR (stool suspension) OR (microflora suspension) OR (fecal flora suspension) OR (fecal donation) OR (fecal microbiota donation) OR (feces donation) OR (stool donation) OR (microflora donation) OR (fecal flora donation) OR (fecal donor) OR (fecal microbiota donor) OR (feces donor) OR (stool donor) OR (microflora donor) OR (fecal flora donor) OR (fecal transfer) OR (fecal microbiota transfer) OR (feces transfer) OR (stool transfer) OR (microflora transfer) OR (fecal flora transfer) OR (fecal infusion) OR (fecal microbiota infusion) OR (feces infusion) OR (stool infusion) OR (microflora infusion) OR (fecal flora infusion) OR (fecal implantation) OR (fecal microbiota implantation) OR (feces implantation) OR (stool implantation) OR (microflora implantation) OR (fecal flora implantation) OR (fecal implant) OR (fecal microbiota implant) OR (feces implant) OR (stool implant) OR (microflora implant) OR (fecal flora implant) OR (fecal instillation) OR (fecal microbiota instillation) OR (feces instillation) OR (stool instillation) OR (microflora instillation) OR (fecal flora instillation) OR (fecal microbiota reconstitution) OR (fecal reconstitution) OR (feces reconstitution) OR (stool reconstitution) OR (microflora reconstitution) OR (fecal flora reconstitution)

### Study selection

Titles, abstracts, and keywords were independently assessed by two investigators (WSN and XMQ) to determine the appropriateness of the publications. Both investigators checked all the articles in accordance with the inclusion criteria and exclusion criteria. Disagreement was resolved by a third investigator (CHL). Original full-text articles, letters to the editor, abstracts of scientific conferences, case reports and case series which were published between 1913 and 2015 were reviewed. Studies involving the AEs of FMT for human sujects of any age were included into this study. Studies evaluating treatments with cultured bacteria other than human feces, animal studies and non-original reports (reviews, systematic reviews, meta-analyses, editorials, etc) were excluded.

### Data Collection and List of Items

Data extraction was conducted according to the above mentioned inclusion and exclusion criteria and cross-checked by the two independent investigators (WSN and XMQ). When publications included patients from a previous study and newly enrolled ones, only the latter were brought into the study. Items of this study were listed as follows: (i) the study characteristics (the first author, the year of publication, the length of follow-up); (ii) the patients (the number, the reason for FMT, the prior therapy); (iii) the relationship between donors and recipients; (iv) the FMT procedure (the patient preparation for FMT, the weight of infused stools, the route of infusion, the number of infusion); (v) the detailed descriptions of AEs (the onset time, the causality between AEs and FMT, how the AEs relieve and the outcomes).

### Definition of causality between the AEs and FMT

The relationship between the AEs and FMT were categorized into four types as previously dsecribed with minor modifications: definitely related, probably related, possibly related, and unrelated to FMT [37].

#### Definitely related

AEs caused by endoscopic procedure during FMT; an event that follows a reasonable temporal sequence from FMT exposure; that follows a known or expected response pattern to the FMT; that is confirmed by stopping the FMT procedure; and that is not explained by any other reasonable hypothesis.

#### Probably related

An event that follows a reasonable temporal sequence from FMT procedure; that follows a known or expected response pattern to the FMT; that is confirmed by stopping the FMT procedure; and that is unlikely to be explained by the known characteristics of the subject’s clinical state or by other interventions.

#### Possibly related

An event that follows a reasonable temporal sequence from FMT procedure; that follows a known or expected response pattern to FMT; but that could readily have been caused by a number of other factors.

#### Unrelated

An event that can be determined with certainty to have no relationship to FMT.

### Definition of severity of AEs

#### Adverse events (AEs)

AE is defined as any untoward medical occurrence in a patient after administration of FMT that does not necessarily have a causal relationship with this treatment. Therefore, an AE can be any unfavorable and unintended sign (including an abnormal laboratory finding), symptom, or disease temporally associated with FMT, whether or not related to the FMT [37].

#### Serious adverse events (SAEs)

A SAE is any adverse experience occurring during or after FMT that results in any of the following outcomes: death, life-threatening experience, inpatient hospitalization or prolongation of existing hospitalization, persistent or significant disability or incapacity, congenital anomaly or birth defect, or an important medical event [37].

## Results

### Included studies

**Fig 1:**
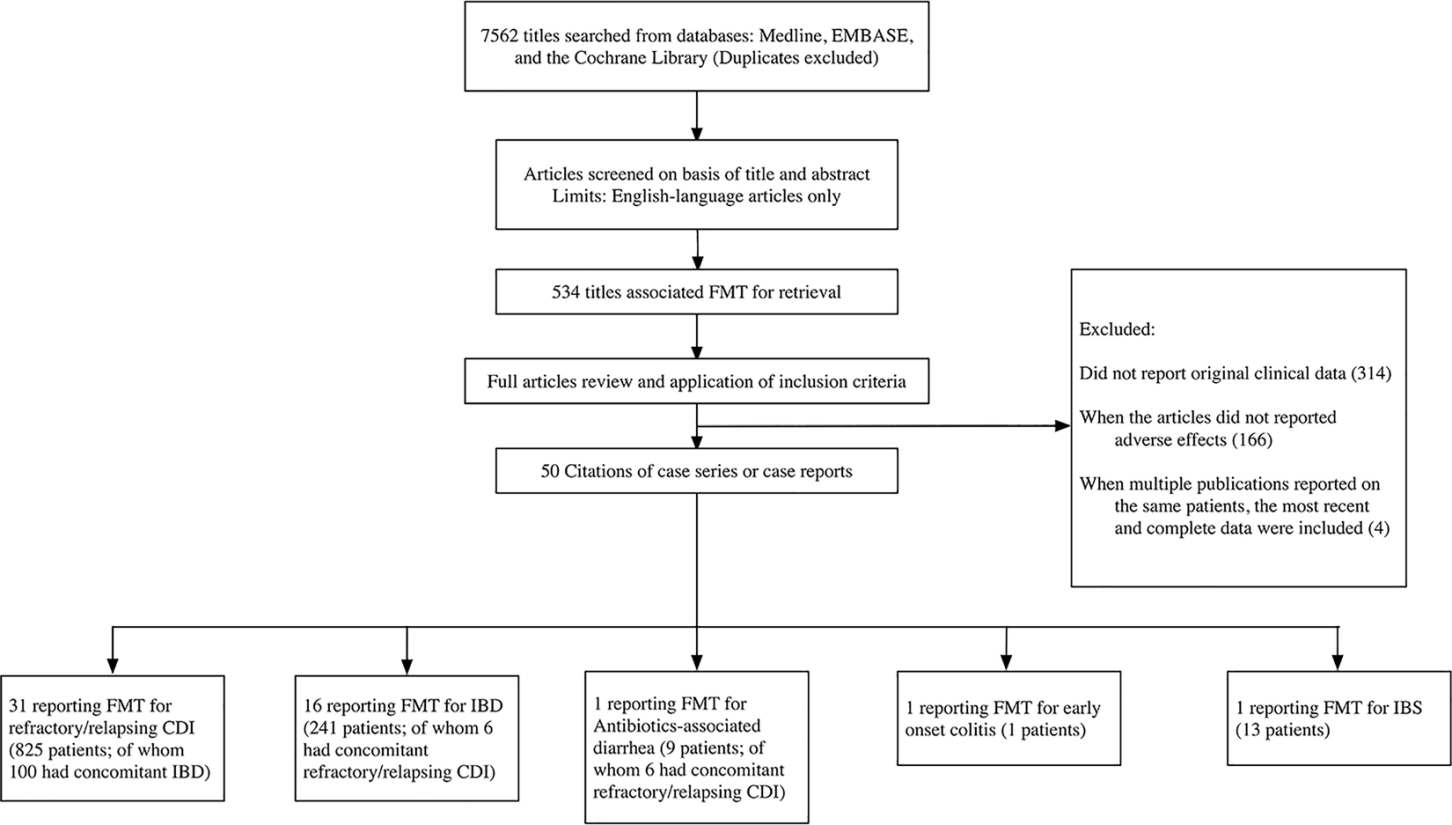
Flow chart of studies of adverse events of fecal microbiota transplantation. FMT: fecal microbiota transplantation, CDI: *Clostridium difficile* infection, IBD: inflammatory bowel disease, IBS: irritable bowel syndrome.

A total of 7562 original articles about FMT were identified. Among them, 534 were selected for further assessment according to the titles. After reviewing the full text articles, 50 of them fulfilled the inclusion criteria among which 16 were case series, 9 were case reports and 4 were randomized controlled trials (Fig 1). The excluded articles were presented in S1 Appendix. Besides, conference abstracts and letters to the editor were included. The included studies were published during the period from 1998 to 2015, with a span of 18 years. The follow-up time after FMT ranged between 2 weeks to 68 months.

### Patient characteristics

In the selected 50 publications, a total of 1089 patients were treated with FMT (age range: 1–95 years). Among them, 831 patients were affected by refractory or recurrent CDI, of whom 106 had concomitant IBD; 235 were affected by independent IBD; 1 affected by early onset colitis; 9 affected by antibiotic-associated diarrhea (AAD), of whom 6 had concomitant CDI; and 13 affected by IBS. Overall, 78 kinds of AEs were reported to happen on 310 patients during or after FMT. The majority of AEs were presented as mild symptoms such as abdominal disomfort, diarrhea, transient fever, nausea, vomiting and constipation. Each AE was cited once for one patient since the AE always recorded only once during a multiple FMT in one course of treatment.

Since AEs usually overlapped, we could not obtain the exact total number of patients with AEs in a publication. Hence, we took the number of patients with the most frequent kind of AEs from each publication for further calculating the overall incidence of AEs. Based on the above mentioned statistical principles, the overall incidence of AEs was 28.5% (310/1089). The incidences of AEs in CDI and non-CDI (IBD, AAD, IBS and early onset colitis) were 28.0% (233/831) and 29.8% (77/258) respectively.

### Causality between AEs and FMT

Many factors could be involved in the development of AEs, including the individual difference of recipients, donors, methods of administration and regimen of FMT. The causality between AEs (including SAEs) and FMT was analyzed according to the description as above [37]. As a result, AEs were described to be attributable to FMT in 42 publications (Tables 2 and 3). Five kinds of AEs were reported to be definitely related to FMT in 5 publications. Thirty-eight kinds of AEs, probably related to FMT, were reported in 35 articles and were considered as results of temporary systemic immune response to the applied bacteria. In addition, 25 kinds of AEs were reported to be possibly related to FMT in 13 articles. Finally, 38 kinds of AEs were reported to be unrelated to FMT in 22 articles (Table 4).

**Table 2:** Attributable adverse events are grouped by route of administration (Upper gastrointestinal routes; Lower gastrointestinal routes).

First Author, Year (Ref.)	AEs	The number of patients with AEs	Sample size	Causality between AEs and FMT	Routes of infusion
**Upper gastrointestinal routes**					
**Vermeire, 2012 [60]**	Fever; Abdominal tenderness	3	4	Probably	Nasojejunal tube
**Cui, 2014 [41]**	Fever; Increased diarrhea	7	30	Probably	Gastroscopy (mid-gut)
**Van Nood, 2013 [61]**	Belching; Nausea; Abdominal cramps; Diarrhea; Abdominal pain; Infection; Dizziness combined with diarrhea; Constipation	27	29	Probably	Nasoduodenal tube
**Aas, 2003 [62]**	Death from peritonitis	1	18	Possibly	Nasogastric tube
**MacConnachie, 2009 [42]**	Upper gastrointestinal hemorrhage	1	15	Possibly	Nasogastric tube
**Kronman, 2015 [63]**	Vomiting; Mucoid stools	1	10	Probably	Nasogastric, nasoduodenal or nasojejunal tube
**Pinn, 2014 [26]**	Flatulence	1	13	Probably	Esophagogastroduodenoscopy
**Wang, 2013 [64]**	Diarrhea	5	16	Probably	Gastroscopy
**Suskind, 2015 [65]**	Rhinorrhea; Sore throat	5	9	Definitely	Nasogastric tube
	Abdominal pain; Bloating; Diarrhea; Flatulence			Probably	
**Suskind, 2015 [66]**	Nasal stuffiness; Flatulence	1	4	Probably	Nasogastric tube
	Bloating			Possibly	
**Rossen, 2015 [50]**	Discomfort tube placement; Fever; Nausea; Malaise; Increase of stool frequency/diarrhea; Headache; Vomited fecal infusion; Vomited bowel prep; Vomiting; Abdominal cramps; Abdominal pain; Abdominal murmurs; Dizziness; Mild constipation	34	48	Probably	Nasoduodenal tube
**Borody, 2003 [67]**	Sore throat	3	8	Definitely	Nasojejunal tube
**Lower gastrointestinal routes**					
**Kump, 2013 [68]**	Fever; Temporary increase of CRP and IL-6; Increase in stool frequency	1	6	Probably	Colonoscopy
**Zhang, 2013 [69]**	Severe cold	1	1	Possibly	Colonoscopy
**Quera, 2013 [44]**	Fever; Bacteremia	1	1	Probably	Colonoscopy
**Kunde, 2013 [70]**	Fever; Chills; Abdominal fullness	2	10	Probably	Enema
	UC flare			Possibly	
**Gustafsson, 1998 [71]**	Diarrhea	3	9	Probably	Enema
**Lee, 2014 [72]**	Transient constipation; Excess flatulence	9	94	Probably	Enema
**Hamilton, 2012 [73]**	Irregularity of bowel movements; Excessive flatulence	14	43	Probably	Colonoscopy
**Khoruts, 2010 [74]**	Constipation; Irregularity of bowel movements	1	1	Probably	Colonoscopy
**Pierog, 2014 [75]**	Appendicitis	1	6	Possibly	Colonoscopy
**Silverman, 2010 [76]**	Urinary tract infections	2	7	Possibly	Enema
**Hohmann, 2014 [39]**	Cytomegalovirus colitis	1	1	Probably	Enema
**De Leon, 2013 [40]**	Transient relapse of UC	1	1	Probably	Colonoscopy
**Schwartz, 2013 [43]**	Norovirus Gastroenteritis	1	13	Probably	Colonoscopy
**Brandt, 2012 [20]**	Peripheral neuropathy; Sjogren’s disease; Idiopathic Thrombocytopenic purpura; Rheumatoid arthritis	4	77	Possibly	Colonoscopy
**Mellow, 2011 [77]**	Relapse of CDI	1	13	Possibly	Colonoscopy
**Mandalia, 2014[78]**	Diverticulitis; Fever	1	1	Probably	Colonoscopy
**Dutta, 2014 [79]**	Fever; Bloating	5	27	Probably	Enteroscopy and colonoscopy
**Ray, 2014 [80]**	Pain/nausea; Bloating/cramps; Gas/nausea	4	20	Probably	Colonoscopy
	Continuing diarrhea			Possibly	
**Satokari, 2015 [53]**	Mild transient fever	2	49	Probably	Colonoscopy
**Sun, 2014 [45]**	Multi-organism bacteremia	1	1	Probably	Colonoscopy
**Mandalia, 2014 [81]**	Abdominal pain	1	29	Probably	Colonoscopy
**Cammarota, 2015 [49]**	Diarrhea; Bloating and abdominal cramping	19	20	Probably	Colonoscopy

**Table 3:** Attributable adverse events are grouped by route of administration (Upper and lower gastrointestinal routes; Not mention of the routes; Capsule).

First Author, Year (Ref.)	AEs	The number of patients with AEs	Sample size	Causality between AEs and FMT	Routes of infusion
**Upper and lower gastrointestinal routes**					
**Angelberger, 2013 [52]**	Sore throat	5	5	Definitely	Nasojejunal tube and enema
	Fever; Temporary increase in CRP; Worsening of diarrhea; Flatulence; Vomiting			Probably	
**Vandelplas, 2014 [38]**	Vomiting; Profuse sweating; Paleness; Tachycardia; Fever	1	1	Probably	Colonoscopy and nasoduodenal tube
**Russell, 2014 [82]**	Mucoid stools; Bloating; Cramping; Loose stools; Abdominal pain; Gassiness; Diarrhea; Blood in stool	3	10	Probably	Colonoscopy and nasogastric tube
**Greenberg, 2013 [83]**	Transient worsening of abdominal distension	3	16	Probably	Colonoscopy and nasojejunal infusion
**Not mention of the routes**					
**Kelly, 2014 [84]**	Death from aspiration; Minor mucosal tear	12	80	Definitely	NR
	Fever; Bloating and abdominal discomfort; Abdominal pain			Probably	
	IBD flare; Self-limited diarrheal illness; Hip pain; Pertussis; Nausea; Death from pneumonia; Diarrhea, encephalopathy and pancytopenia; Colectomy			Possibly	
**Brandt, 2013 [85]**	Transient abdominal distension with bloating	2	12	Probably	NR
**Wilson, 2014 [86]**	Diarrhea or nullloose stoolnull; Bloating; Flatus; Constipation; Abdominal pain; GERD (gastroesophageal reflux disease)	12	45	Probably	NR
**Obi, 2014 [87]**	Bowel perforation	1	20	Definitely	NR
**Borody, 2003 [67]**	Flatulence; Rectal discomfort; Nausea; Abdominal cramping; Bloating; Headache; Abdominal pain	7	24	Probably	Combination of colonoscopy and/ or rectal enema and/or nasojejunal tube
**Capsule**					
**Youngster, 2014 [59]**	Abdominal cramping and bloating	6	20	Possibly	Capsule

**Table 4:** Adverse events are grouped by their causality with fecal microbiota transplantation.

First Author, Year (Ref.)	Definitely related to FMT	Probably related to FMT	Possibly related to FMT	Unrelated to FMT
**Vermeire, 2012 [60]**		Fever; Abdominal tenderness; Transient increase of CRP		
**Kump, 2013 [68]**		Fever; Temporary increase of CRP and IL-6; Increase in stool frequency		
**Angelberger, 2013 [52]**	Sore throat	Fever; Temporary increase in CRP; Worsening of diarrhea; Flatulence; Vomiting	Itchiness; Erythema; Paresthesia of the hip; Blisters on the tongue	Common cold; Unexplained pancreatitis; Collapse due to orthostatic disorder
**Zhang, 2013 [69]**			Severe cold	
**Cui, 2014 [41]**		Fever; Increased diarrhea		
**Quera, 2013 [44]**		Fever; Bacteremia		
**Kunde, 2013 [70]**		Fever; Chills; Abdominal fullness; Lower back pain; Nausea; Vomiting	Headache, UC flare	Cervical lymphadenopathy
**Vandelplas, 2014 [38]**		Vomiting; Profuse sweating; Paleness; Tachycardia; Fever		
**Russell, 2014 [82]**		Mucoid stools; Bloating; Cramping, Loose stools; Abdominal pain; Gassiness; Diarrhea; Blood in stool		
**Van Nood, 2013 [61]**		Belching; Nausea; Abdominal cramps; Diarrhea; Abdominal pain; Infection; Dizziness combined with diarrhea; Constipation		Symptomatic choledocholithiasis
**Gustafsson, 1998 [71]**		Diarrhea		
**Lee CH, 2014 [72]**		Transient constipation; Excess flatulence		
**Hamilton, 2012 [73]**		Irregularity of bowel movements; Excessive flatulence		
**Khoruts, 2010 [74]**		Constipation; Irregularity of bowel movements		
**Kelly, 2014 [84]**	Death from aspiration; Minor mucosal tear	Fever; Bloating; Abdominal discomfort;Abdominal pain post FMT colonoscopy	IBD flare; Self-limited diarrheal illness; Hip pain; Pertussis; Nausea; Death from pneumonia; Diarrhea; Encephalopathy and pancytopenia; Colectomy	Cerebrovascular accident, nausea and vomiting; Fall and sustained hip fracture; Influenza and diarrhea (non-CDI); Catheter infection
**Pierog, 2014 [75]**			Appendicitis	
**Youngster, 2014 [59]**		Infectious irritable bowel symptoms		Relapse of severe CDI
**Silverman, 2010 [76]**			Urinary tract infections;	
**Hohmann, 2014 [39]**		Irregularity of bowel movements; Weakness; Fatigue; Decreased appetite; Abdominal pain; Night sweats; Fever; Cytomegalovirus colitis	Minor joint pains; Weight loss	
**De Leon, 2013 [40]**		Transient relapse of UC		
**Schwartz, 2013 [43]**		Norovirus Gastroenteritis		
**Brandt, 2012 [20]**			Peripheral neuropathy; Sjogren ‘ s disease; Idiopathic Thrombocytopenic purpura; Rheumatoid arthritis	Death for unknown causes, metastatic colon cancer, metastatic ovarian cancer, pneumonia, myocardial infarction, cerebral vascular accident and sepsis
**Mellow, 2011 [77]**			CDI relapse	Death from pneumonia; Death from superior mesenteric vein thrombosis; Death from ovarian cancer
**Aas, 2003 [62]**			Death from peritonitis	Death from pneumonia
**Mandalia, 2014 [78]**		Diverticulitis; Fever		
**MacConnachie, 2009 [42]**			Upper gastrointestinal hemorrhage	
**Kronman, 2015 [63]**		Vomiting; Mucoid stools		
**Dutta, 2014 [79]**		Low-grade fever; Bloating		
**Friedman-Moraco, 2014 [88]**				Cerebral vascular event; Bronchiolitis obliterans
**Ray, 2014 [80]**		Pain/nausea; Bloating/cramps; Vomiting; Abdominal pain	Continuing diarrhea	Cerebrovascular accident
**Pinn, 2014 [26]**		A transient increase in flatus		
**Mattila, 2012 [19]**				Died of unrelated illnesses
**Satokari, 2015 [53]**		Mild transient fever		
**Trubiano, 2013 [89]**				Renal failure, episodes of VAP (ventilator-associated pneumonia) and death
**Garborg, 2010 [90]**				Died of serious co-morbid conditions
**Borody, 2003 [67]**	Sore throat	Flatulence, rectal discomfort, nausea, abdominal cramping, bloating, headache, abdominal pain		
**Sun, 2014 [45]**		Multi-organism bacteremia		
**Mandalia, 2014 [81]**		Abdominal pain		
**Greenberg, 2013 [83]**		Transient worsening of abdominal distension		
**Brandt, 2013 [85]**		Transient abdominal distension with bloating		
**Fischer, 2014 [91]**				Multi-organ failure
**Wilson, 2014 [86]**		Diarrhea; Bloating; Flatus; Constipation; Abdominal pain; GERD (gastroesophageal reflux disease)		Infections; recurrent CDI; Death of lung cancer
**Wang, 2013 [64]**		Diarrhea		
**Fischer, 2014 [92]**				Refractory CD, refractory CDI, UC flare, non-infectious severe diarrhea, recurrent CDI and worsening CD
**Obi, 2014 [87]**	Bowel perforation			Diarrhea
**Suskind, 2015 [65]**	Rhinorrhea, sore throat	Abdominal pain, bloating, diarrhea, flatulence		Mild stuffy nose,
**Suskind, 2015 [66]**		Nasal stuffiness, flatulence	Bloating	Vomiting, developed *C difficile* diarrhea
**Cammarota, 2015 [49]**		Diarrhea, bloating and abdominal cramping		
**Rossen, 2015 [50]**		Transient borborygmia, increase of stool frequency, vomiting, transient fever,		Suspicion of a small bowel perforation, cytomegalovirus infection, abdominal pain, cervix carcinoma
**Moayyedi, 2015 [56]**				Crohn’s colitis, active ulcerative colitis, *Clostridium difficile* toxin positive

#### Donors and AEs

So far, there is no unified standard to screen the stool samples from donors. The following donor screening tests were applied to the donors in the selected 50 publications: viral screenings (hepatitis A virus, hepatitis B virus, hepatitis C virus, Epstein–Barr virus, human immunodeficiency virus, treponema pallidum, and cytomegalovirus), stool tests for *Clostridium difficile* toxin, and routine bacterial culture for enteric pathogens (*Escherichia coli*, *Salmonella*, *Shigella*, *Yersinia*, *Campylobacter*), parasites and ova. However, the donors who were in the latent period of infection could not be excluded by the above screening tests and thus these donors might contribute to the development of infectious AEs.

In addition, individual differences of donors may also lead to AEs. For example, one patient (a 1-year-old girl) developed fever, vomiting and tachycardia after receiving the fecal transplant from her brother, while she well tolerated the transplant from her niece [38]. For the infrequent infection of cytomegalovirus in FMT receipt, it may be caused by the donors with young age [39]. Finally unrecognized pathogens that were carried by donors might induce AEs [40].

Related donors (family members) for FMT were reported in 11 publications. Unrelated donors were reported in 9 publications.

#### Preparation and route of administration and AEs

For FMT via upper gastrointestinal routes, the recipients were prepared in fasting condition. For FMT via lower gastrointestinal routes, bowel lavage and/or antibiotics were given to the recipients before FMT. However, no association of AEs with the preparation of FMT was found in the 50 publications. Of note, in Bota Cui’s report, the recipients who took metoclopramide before FMT manifested fewer AEs, suggesting metoclopramide might potentially help avoid the AEs [41].

The routes of administration are listed as follows according to the frequency that they were used: colonoscopy (26 publications), retention enema (8 publications), nasogastric tube (6 publications), nasojejunal tube (5 publications), gastroscopy (2 publications), sigmoidoscopy (1 publication), nasoduodenal tube (4 publications), enteroscopy (1 publication), esophagogastroduodenoscopy (1 publication) and capsule (1 publication). Among the above routes of administration, lower gastrointestinal routes include colonoscopy, sigmoidoscopy and retention enema. Upper gastrointestinal routes include the remaining means. Compared with upper gastrointestinal routes, lower gastrointestinal routes were more widely used. After exclusion of the publications in which the routes of administration were not clearly stated, the proportion of patients affected by FMT-attributable AE is 43.6% (89/204) for upper gastrointestinal routes of FMT administration, while the incidence dropped to 17.7% (76/430) for lower gastrointestinal routes. The FMT-attributable AEs were grouped by routes of administration (Tables 2 and 3).

Among the 78 kinds of AEs, 5 kinds were definitely related to endoscopic manipulation. Of these, nasal stuffiness, sore throat, rhinorrhea and upper gastrointestinal hemorrhage happened on a total of 8 patients in 4 publications, which were attributable to upper gastrointestinal routes administration. It seems that patients are likely to be injured by invasive endoscope procedures for upper gastrointestinal routes of FMT administration.

The commonest attributable AE was abdominal discomfort for both upper and lower gastrointestinal routes, including abdominal pain, increased stool frequency, flatulence, bloating, cramps and other nonspecific symptoms. For upper gastrointestinal routes of administration in 12 publications, 29.9% (61/204) patients (in 9 publications) were reported to suffer abdominal discomfort after FMT. For lower gastrointestinal routes in 22 publications, 13.0% (56/430) patients (in 10 publications) developed abdominal discomfort after FMT. The upper gastrointestinal routes were therefore more likely to develop abdominal discomfort compared with lower gastrointestinal. The second commonest attributable AE was transient fever which was happened on 3.4% (7/204) and 2.8% (12/430) patients for upper and lower gastrointestinal routes of FMT administration, respectively (Table 2).

### Classification of AEs based on severity

Mild to moderate AEs such as abdominal pain, abdominal cramping, flatulence, increased stool frequency, constipation, vomiting, belching, fever and transient increase of C-reactive protein (CRP) were reported in most of the selected 50 publications and ususally did not cause critical clinical outcome. Hence, we paid emphatic attention to SAEs and listed 44 kinds of SAEs that were reported in 27 publications (Table 5), of which 18 kinds were associated with FMT procedure. Totally 9.2% (100/1089) patients developed SAEs. The incidences of SAEs were 2.0% (4/196) and 6.1% (40/659) for upper and lower gastrointestinal routes respectively, which suggest that lower gastrointestinal routes of FMT administration induce more SAEs compared with upper routes.

**Table 5:** Serious adverse events (SAEs) of fecal microbiota transplantation.

First Author, Year (Ref.)	The total number of patients	Patient Preparation to FMT	Infused Stools	Route of Infusion	Donor Relation-ship	Number of Infusion	SAE		Causality between AEs and FMT	Day post-FMT event occurred	How to relieve the AE	Follow-Up
**De Leon, 2013 [40]**	1 UC/CDI	Antibiotics	600ml infusion	Colonoscopy	Related	1	Transient relapse of UC		Probably	9 days	Prednisone, mesalamine	2 weeks
**Hohmann, 2014 [39]**	1 UC	NR	NR	Home FMT	Related	4	Cytomegalovirus colitis		Probably	Several weeks	Anti- cytomegalovirus therapy	NR
**Van Nood, 2013 [61]**	16CDI	Bowel lavage	500ml infusion	Nasoduodenal tube	Unrelated	1 or 2	Symptomatic choledocholithiasis		Unrelated	During follow-up	Stone extraction	15 weeks
**Schwartz, 2013 [43]**	13 CDI	Antibiotics, Bowel preparation	NR	Colonoscopy	Related	NR	Norovirus Gastroenteritis;		Probably	2 days	Self-limited	NR
							Norovirus Gastroenteritis; Relapse of CDI		Unrelated	12 days		
**Brandt, 2012 [20]**	77 CDI	Antibiotics, Bowel preparation	300–700ml infusion	Colonoscopy	Related/Unrelated	1 or 2	Peripheral neuropathy		Possibly	NR	NR	3–68 months
							Sjogren ‘ s disease					
							Idiopathic thrombocytopenic purpura					
							Rheumatoid arthritis					
							Died of unrelated diseases					
**Mellow, 2011 [77]**	13 CDI	NR	300–600 ml infusion	Colonoscopy	NR	1	Death	B strep pneumonia	Unrelated	1 month	Died	1–10 months
								Superior mesenteric vein thrombosis	Unrelated	5 months		
								Ovarian cancer	Unrelated	7 months		
							Relapse of CDI		Unrelated	7 months	Relapse of CDI	
**Youngster, 2014 [59]**	20 CDI	NR	650μl*15	Capsule	Unrelated	1 or 2	Relapse of severe CDI		Unrelated	NR	Receiving the remaining 15 capsules	8 weeks
**Aas, 2003 [62]**	18 recurrent *Clostridium difficile* Colitis	NR	≤30 g	Nasogastric tube	NR	1	Death	Peritonitis	Possibly	3 days	Died	90 days
								Pneumonia	Unrelated	14 days		
**Kunde, 2013 [70]**	10 UC	NR	70–113 g	Enema	Related and unrelated	5	UC flare		Possibly	Third week	Corticosteroid enema	1 month
**Kelly, 2014 [84]**	80 CDI in Immunoco-mpromised Patients	NR	NR	Colonoscopy or others	NR	1 or more	Death	Pneumonia	Possibly	13 days	Died	3–46 months
								Aspiration	Definitely	1 day		
							Hospitalizations	Fever, diarrhea, encephalopathy and pancytopenia	Possibly	4 days	NR	
								Abdominal pain post FMT colonoscopy	Probably	0 day	Self-limited	
								IBD flare	Possibly	< 84 days	NR	
								Cerebrovascular accident; nausea and vomiting	Unrelated	21 days		
								Colectomy	Possibly	< 28 days		
								Fall and sustained hip fracture	Unrelated	84 days		
								Influenza B and diarrhea (non-CDI)	Unrelated	3 days		
								Catheter infection	Unrelated	14 days		
**Mandalia, 2014 [78]**	1 CDI/CD	NR	100g	Colonoscopy	NR	1	Diverticulitis, fever		Probably	2–3 hours	Antibiotics	3 months
**Quera, 2013 [44]**	1 CD/CDI	NR	NR	Colonoscopy	NR	1	Bacteriemia		Probably	24 hours	Aztreonam	5 months
**Pierog, 2014 [75]**	6 CDI	Bowel lavage	250–500 mL infusion	Colonoscopy	Related	1	Appendicitis		Possibly	2 weeks	Appendectomy	12 weeks
**Silverman, 2010 [76]**	7 CDI	Stop anti-CDI antimicrobial	50 mL infusion	Enema	Related	1 or 2	Post infectious irritable bowel symptoms		Unrelated	NR	Cotrimoxazole	14 months
							Urinary tract infections				Ampicillin/ gentamicin and ciprofloxacin	
**Friedman-Moraco, 2014 [88]**	2 CDI	NR	80mL	Nasojejunal tube	Related	2	Cerebral vascular event		Unrelated	NR	NR	1 year
			250mL	Colonoscopy								
			325mL	Colonoscopy	Unrelated	2	Bronchiolitis obliterans and death			5 days		NR
			100mL	Nasojejunal tube								
**Ray, 2014 [80]**	20 CDI	Stop all antibiotics	NR	Colonoscopy	Related and unrelated	1	Cerebrovascular accident		Unrelated	> 1 month	NR	3 months
**Mattila, 2012 [19]**	70 CDI	Antibiotics were stopped Colonic lavage	100mL suspension	Colonoscopy	Related and unrelated	1 or more	Died of unrelated illnesses		Unrelated	Within 1 year	NR	1 year
							No response and death		Unrelated	Within 3 months		
**Trubiano, 2013 [89]**	1 CDI	NR	30mL suspension	Nasogastric tube	Related	1	Renal failure, episodes of ventilator-associated pneumonia and death		Unrelated	NR	Continuous renal replacement therapy	NR
**Garborg, 2010 [90]**	40 recurrent *Clostridiumdifficil*e-associated diarrhea	Fast	50–100 g	Gastroscopy or colonoscopy	Related and unrelated	1 or 2	Wegener ‘ s granulomatosis, acute myelogenous leukaemia, advanced cardiovascular disease developed fulminant colitis and underwent subtotal colectomy		Unrelated	3 weeks– 2 months	Died	2 months
**Sun, 2014 [45]**	1 CDI	NR	NR	Colonoscopy	NR	NR	Multi-organism bacteremia		Probably	NR	Ampicillin/sulbactam; vancomycin	NR
**Fischer, 2014 [91]**	17 CDI	Bowel preparation	NR	Colonoscopy	Related and unrelated	1, 2 or 3	Multi-organ failure and death	Immunosuppression	Unrelated	NR	NR	NR
								Septic shock				
**Wilson, 2014 [86]**	45 CDI	NR	NR	NR	NR	NR	Infections; Recurrent CDI; Death of lung cancer		Unrelated	NR	NR	6 months
**Fischer, 2014 [92]**	41 CDI/IBD (21 CD, 19UC, 1 indeterminate colitis)	NR	NR	Colonoscopy or Sigmoidoscopy	NR	1 or 2	Refractory CD, refractory CDI, UC flare, non-infectious severe diarrhea, recurrent CDI and worsening CD		Unrelated	NR	NR	NR
**Obi, 2014 [87]**	20 CDI	NR	NR	NR	NR	NR	Bowel perforation		Definitely	NR	Colectomy	4 months
**Suskind, 2015 [66]**	4 UC	Omeprazole, rifaximin, MiraLAX and bowel preparation	Infusion of 30 mL	Nasogastric tube	NR	1	Developed *C difficile* diarrhea		Unrelated	3 months	Vancomycin	6 months
										4 months	NR	
**Rossen, 2015 [50]**	50 UC	Bowel lavage	120 g	Nasoduodenal tube	Unrelated	2	Suspicion of a small bowel perforation		Unrelated	5 weeks	Antibiotics	12 weeks
							Cytomegalovirus infection			7 weeks	Ganciclovir	
							Abdominal pain			11 weeks	Spontaneous recovery	
							Cervix carcinoma			6 weeks	Operation	
**Moayyedi, 2015 [56]**	75 UC	NR	50 mL	Retention enema.	Unrelated	6	Three patients had their diagnoses changed to Crohn’s colitis from ulcerative colitis.		Unrelated	NR	NR	12 months
							Active ulcerative colitis			Three weeks	Urgent colectomy	
							*Clostridium difficile* toxin positive			After study exit	NR	

The FMT-attributable (definitely, probably and possibly related) SAEs included death, pathogen infections, IBD flare, auto-immune diseases, and FMT procedure related injury, etc, while the FMT unrelated SAEs covered death or hospitalization caused by underlying conditions. The commonest SAEs were death, severe infections and relapse of CDI and IBD.

As the most devastating SAEs, death happened on 38 patients in 10 publications (Table 6) and the mortality rate was 3.5% (38/1089). Of these deaths, 1 was definitely related, 2 were possibly related, and 35 were unrelated to FMT. The death that was definitely related to FMT was caused by aspiration during sedation of colonoscopy [42]. The other two deaths were associated with infections which might be outcomes of either FMT procedures or underlying immunocompromised status. Except for the above 3 patients, no evidence supported the notion that the remaining deaths could have been caused or facilitated by preparation, route of infusion, donor, number of infusion or the FMT procedure.

**Table 6:** Summary of death after fecal microbiota transplantation.

First Author, Year (Ref.)	The total number of patients	Patient Preparation to FMT	Infused Stools	Route of Infusion	Donor Relationship	Number of Infusion	Cause of death	Causality between AEs and FMT	Day post-FMT event occurred	Follow-Up
**Garborg, 2010 [90]**	40 recurrent Clostridiumdifficile-associated diarrhea	Fast	50–100 g	Gastroscopy or colonoscopy	Related and unrelated	1 or 2	Wegener ‘ s granulomatosis, acute myelogenous leukaemia, advanced cardiovascular disease developed fulminant colitis and underwent subtotal colectomy	Unrelated	3 weeks– 2 months	2 months
**Mellow, 2011 [77]**	13 CDI	NR	300–600 ml infusion	Colonoscopy	NR	1	B strep pneumonia	Unrelated	1 month	1–10 months
							Superior mesenteric vein thrombosis	Unrelated	5 months	
							Ovarian cancer	Unrelated	7 months	
**Aas, 2003 [62]**	18 recurrent *Clostridium difficile* Colitis	NR	≤30 g	Nasogastric tube	N46R	1	Peritonitis	Possibly	3 days	90 days
							Pneumonia	Unrelated	14 days	
**Kelly, 2014 [84]**	80 CDI in Immunocom-promised Patients	NR	NR	Colonoscopy or others	NR	1 or more	Pneumonia	Possibly	13 days	3–46 months
							Aspiration	Definitely	1 day	
**Friedman-Moraco, 2014 [88]**	2 CDI	NR	325mL	Colonoscopy	Unrelated	2	Bronchiolitis obliterans	Unrelated	5 days	NR
			100mL	Nasojejunal tube						
**Mattila, 2012 [19]**	70 CDI	Antibiotics were stopped, colonic lavage	100mL suspension	Colonoscopy	Related and unrelated	1 or more	Unrelated illnesses	Unrelated	Within 1 year	1 year
							No response and died	Unrelated	Within 3 months	
**Brandt, 2012 [20]**	77 CDI	Antibiotics, bowel preparation	300–700ml infusion	Colonoscopy	Related/Unrelated	1 or 2	Death for unknown causes, metastatic colon cancer, metastatic ovarian cancer, pneumonia, myocardial infarction, cerebral vascular accident and sepsis	Unrelated	NR	3–68 months
**Trubiano, 2013 [89]**	1 CDI	NR	30 mL	Nasojejunal tube	Related	1	Renal failure, episodes of ventilator-associated pneumonia	Unrelated	NR	NR
**Fischer, 2014 [92]**	17 CDI	Bowel preparation	NR	Colonoscopy	Related and unrelated	1 or 2 or 3	Immunosuppression	Unrelated	NR	NR
							Septic shock			
**Wilson, 2014 [86]**	45 CDI	NR	NR	NR	NR	NR	Lung cancer	Unrelated	NR	6 months

Twenty-seven patients were reported to be hospitalized or die for infection in 12 publications (CDI was not included) (Table 7). The incidence of severe infection was 2.5% (27/1089). Among the 27 cases of severe infection, 8 cases were probably or possibly related to FMT and the remaining 19 cases were unrelated to FMT.Out of the 8 cases of severe infection, 2 were viral infection, 2 were bacteriemia infection, and the remaining 4 were infection of unknown pathogens. The pathogens that caused the 2 cases of viral infection were cytomegalovirus [39] and norovirus [43] respectively and the pathogens that caused the 2 cases of bacteriemia infection were *Escherichia coli*, *Proteus mirabilis*, *Citrobacter koseri*, and *Enterococcus faecium* [44, 45]. The cytomegalovirus infection happened after home FMT and was suspected to be probably related to a child donor without strict donor screening. The noroviurs infection was speculated to be probably related to environmental contamination by an endoscopy suite employee. IBD flare happened on 7 patients (4 UC and 3 CD) post-FMT in the 50 selected publications (Table 8) and its incidence reached 0.6% (7/1089). Most patients who suffered from IBD flare were those with low immunity, such as kids, aged people and immunocompromised ones. Of note, patients with IBD flare were administered FMT via lower gastrointestinal routes, such as colonoscopy, sigmoidoscopy or enema. Therefore, IBD flare should arouse attention when FMT was administered via lower gastrointestinal routes. So far, the association between donors and IBD flare has not been defined, for some unrecognized pathogens from donors’ stool might also lead to IBD flare.

**Table 7:** Summary of infections after fecal microbiota transplantation.

First Author, Year (Ref.)	The total number of patients	Patient Preparation to FMT	Infused Stools	Route of Infusion	Donor Relationship	Number of Infusion	SAE (infections)	Causality between AEs and FMT	Day post-FMT event occurred	How to relieve the AE	Follow-Up
**Hohmann, 2014 [39]**	1 UC	NR	NR	Home FMT	Related	4	Cytomegalovirus colitis	Probably	Several weeks	Anti- cytomegalovirus therapy	NR
**Schwartz, 2013 [43]**	13 CDI	Antibiotics, Bowel preparation	NR	Colonoscopy	Related	NR	Norovirus Gastroenteritis	Probably	2 days	Self-limited	NR
							Norovirus Gastroenteritis	Unrelated	12 days		
**Mellow, 2011 [77]**	13 CDI	NR	300–600 ml infusion	Colonoscopy	NR	1	B strep pneumonia	Unrelated	1 month	Died	1–10 months
**Aas, 2003 [62]**	18 recurrent *Clostridium difficile* Colitis	NR	≤30 g	Nasogastric tube	NR	1	Peritonitis	Possibly	3 days	Died	90 days
							Pneumonia	Unrelated	14 days		
**Kelly, 2014 [84]**	80 CDI in Immunocom-promised Patients	NR	NR	Colonoscopy or others	NR	1 or more	Died of pneumonia	Possibly	13 days	Died	3–46 months
							Influenza B and diarrhea (non-CDI)	Unrelated	3 days	NR	
							Catheter infection	Unrelated	14 days		
**Mandalia, 2014 [78]**	1 CDI/CD	NR	100g	Colonoscopy	NR	1	Diverticulitis, fever	Probably	2–3 hours	Antibiotics	3 months
**Quera, 2013 [44]**	1 CD/CDI	NR	NR	Colonoscopy	NR	1	Bacteriemia	Probably	24 hours	Aztreonam	5 months
**Pierog, 2014 [75]**	6 CDI	Bowel lavage	250–500 mL infusion	Colonoscopy	Related	1	Appendicitis	Possibly	2 weeks	Appendectomy	12 weeks
**Silverman, 2010 [76]**	7 CDI	Stop anti-CDI antimicrobial	50 mL infusion	Enema	Related	1 or 2	Post infectious irritable bowel symptoms	Unrelated	NR	Cotrimoxazole	14 months
							Urinary tract infections			Ampicillin/ gentamicin and ciprofloxacin	
**Sun, 2014 [45]**	1 CDI	NR	NR	Colonoscopy	NR	NR	Multi-organism bacteremia	Probably	NR	Ampicillin/sulbactam; vancomycin	NR
**Wilson, 2014 [86]**	45 CDI	NR	NR	NR	NR	NR	HCV seroconversion, urinary tract infection, viral upper respiratory infection, foot infection, eye infection, and shingles	Unrelated	NR	NR	6 months
**Rossen, 2015 [50]**	50 UC	Bowel lavage	120 g	Nasoduodenal tube	Unrelated	2	Cytomegalovirus infection	Unrelated	7 weeks	Ganciclovir	12 weeks

**Table 8:** Summary of relapse of inflammatory bowel disease or *Clostridium difficile* infection after fecal microbiota transplantation.

SAE	First Author, Year (Ref.)	The total number of patients	Patient Preparation to FMT	Infused Stools	Route of Infusion	Donor Relationship	Number of Infusion	Causality between SAEs and FMT	Day post-FMT event occurred	How to relieve the AE	Follow-Up
**Relapse of CDI**	**Schwartz, 2013 [43]**	13 CDI	Antibiotics, Bowel preparation	NR	Colonoscopy	Related	NR	Unrelated	12 days	NR	NR
	**Mellow, 2011 [77]**	13 CDI	NR	300–600 ml infusion	Colonoscopy	NR	1	Unrelated	7 months	NR	1–10 months
	**Youngster, 2014 [59]**	20 CDI	NR	650μl*15	Capsule	Unrelated	1 or 2	Unrelated	NR	Receiving the remaining 15 capsules	8 weeks
	**Wilson, 2014 [86]**	45 CDI	NR	NR	NR	NR	NR	Unrelated	NR	NR	6 months
	**Suskind, 2015 [66]**	4 UC	Omeprazole, rifaximin, MiraLAX and bowel preparation	Infusion of 30 mL	Nasogastric tube	NR	1	Unrelated	3 months	Vancomycin	6 months
									4 months	NR	
**Relapse of IBD**	**De Leon, 2013 [40]**	1 UC/CDI	Antibiotics	600ml infusion	Colonoscopy	Related	1	Probably	9 days	Prednisone, mesalamine	2 weeks
	**Kunde, 2013 [70]**	10 UC	NR	70–113 g	Enema	Related and unrelated	5	Possibly	Third week	Corticosteroid enema	1 month
	**Kelly, 2014 [84]**	80 CDI in Immunocomprom-ised Patients	NR	NR	Colonoscopy or others	NR	1 or more	Possibly	< 84 days	NR	3–46 months
**Relapse of UC, relapse of CDI**	**Fischer, 2014 [92]**	41 CDI/IBD (21 CD, 19UC, 1 indeterminate colitis)	NR	NR	Colonoscopy or Sigmoidoscopy	NR	1 or 2	Unrelated	NR	NR	NR

Although unrelated to FMT, CDI relapse was still another unignorable SAE, the incidence of which reached 0.9% (10/1089) in the selected 50 publications (Table 7).

## Discussion

The human gastrointestinal tract harbors the largest number of microbes in the human body, which is referred to as the gut microbiota. Perturbations in the gut microbiota have been associated with conditions as diverse as gastrointestinal diseases and even systemic disorders [46]. As a microbiota-targeted therapy, FMT shows promise in controlling bacteria-associated disorders, especially recurrent or refractory CDI. Nevertheless, with the growing application of FMT, safety evaluation for FMT is increasingly urgent and potential risks of FMT must be paid attention to. Previous studies focus on the effectiveness of FMT treatment on CDI [47], IBD [25] and other digestive and nondigestive disorders [23, 48], lacking of emphasizing AEs of FMT. Landy et al [48] reviewed publications about FMT therapy for gastrointestinal diseases that were published before 2011 and did not find any reports regarding FMT related AEs. A more recent systematic review about FMT for IBD treatment (2012) summarized AEs that were reported in just three citations [25]. Lately, two randomized controlled trials of FMT for CDI [49] and UC [50] reported a high incidence of AEs, suggesting that under-reporting AEs of FMT may exist in many other cases. Therefore, there is an urgent need to systematically review and analyze the characteristics of AEs of FMT to evaluate the safety of the procedure.

Here, we selected 50 publications in which AEs of FMT were reported. Totally 78 kinds of AEs happened on 310 patients following FMT treatment. The AEs of FMT were divided into two major categories, namely related and unrelated to FMT. Our analytical results showed that the AEs related to FMT (including definitely, probably and possibly related) accounted for a larger proportion than the AEs unrelated to FMT. Moreover, we found that SAEs related to FMT such as death, viral and bacterial infections, transient relapse of IBD, were not rare and therefore deserved attention and consideration in the procedure of FMT.

Human Microbiome Project (HMP) has sampled the microbiome of many people to get a better idea of variability, and how microbes work together in complex communities. HMP implied that because the microbiome is more varied than the genome, and easier to modify, it gives a more logical starting point for individual treatments [51]. As observed by Angelberger et al, most AEs may be caused by the applied bacteria into the gut [52], which, in our opinion, could be further supported by the notion that most patients receiving FMT were under the conditions of impaired intestinal mucosal barrier and severe inflammation. In a recent observational cohort study of FMT for treating recurrent CDI, mild transient fever happened on two patients receiving FMT [53]. The authors speculated that FMT itself rather than glycerol used in the frozen preparations caused the AE, which was in agreement with Angelberger’s opinion. Though all donors underwent blood and stool tests before FMT as recommended by FDA, some unrecognized infective agents might cause AEs of FMT. Since variability in donor microbiotas exists, it is necessary to establish a better donor screening methodologies. Moreover, the inclusion criteria of FMT donors for recurrent or refractory CDI have been established, but an agreement of the inclusion criteria of donors for IBD, IBS, metabolic diseases, and other extra-CDI have not been reached, which might engender potential risks for AEs of FMT [54, 55]. A recent placebo-controlled randomized trial demonstrated that FMT with the donation of two donors were more effective but with milder AEs than the other donors. Sequencing analysis of the microbiota was conducted for the two donors and they turned out to have similar taxonomic profiles [56]. Previous study also demonstrated that genetic variation in immune genes could result in variability in susceptibility to enteric infection in germfree mice [57]. Thus, genetic variation may paly a key role in variability in microbiota composition, susceptibility to enteric infection, response to FMT treatments, and even AEs. The emerging metagenomics, genetic and microbiota screening methodologies could be useful for identifying better donor sources for FMT therapies in the future [58].

Route of fecal infusion is another concern in FMT that may lead to AEs. Lower gastrointestinal routes, including colonoscopy, sigmoidoscopy, and retention enema, were more widely used than upper gastrointestinal routes. We found that the patients who received FMT treatment via upper gastrointestinal routes were more likely to develop AEs than those who received FMT treatment via lower gastrointestinal routes (43.9% vs. 20.6%). To avoid injury associated AEs during endoscopic process, noninvasive and patient-acceptable routes can be chosen for FMT treatment. Actually, a recent pilot study in which frozen capsules FMT was administered orally for patients with recurrent CDI demonstrated a high incidence of diarrhea resolution (overall 90%) but few mild AEs such as abdominal cramping and bloating [59]. Therefore, capsules would potentially make FMT procedure safer by avoiding procedure-related complications as well as availability for long-term usage. Furthermore, the encapsulated FMT can be accessible to a wider range of patients, especially to those who cannot withstand the endoscopic procedure. Hence, large randomized controlled studies for the safety and therapeutic efficacy of encapsulated FMT are warranted.

Though this systematic review provides a handful of valuable messages for clinical application of FMT, some limitations need to be addressed. First, the incidences of AEs might be underestimated. On one hand, since AEs usually overlapped, we took the number of patients with the most frequent kind of AE from a publication for further calculating the overall incidence of AEs. On the other hand, transient or mild AEs were sometimes ignored by researchers, resulting in the missing data of AE occurrence. Secondly, some potential confounding factors such as the health conditions of the donors, the time span from FMT exposure to the onset of the AEs and the outcomes of AEs could have substantial impacts on the classification of AEs. Thirdly, there was subjective nature in the classification of AEs. In most instances, it is impossible for us to obtain the original data of the publications we selected. Therefore we categorized AEs according to the authors’ subjective description or FDA general definitions of AEs for drugs or therapy [37].

## Conclusion

Though FMT was validated to be a beneficial therapeutic strategy, we should pay enough attention to AEs of FMT. In order to prevent or treat AEs during or after FMT, more clinical trials and fundamental research are urged to elucidate the exact mechanism of how FMT causes AEs and set up a guideline on how to handle FMT-related AEs in different situations.

## Supporting Information

S1 Appendix Full-text excluded articles. (DOCX)

S2 Appendix PRISMA Checklist. (DOC)

S3 Appendix PRISMA flow diagram. (DOC)
