# Supplementary material for: Systematic Review: Adverse Events of Fecal Microbiota Transplantation
Source: PLoS One. 2016 Aug 16;11(8):e0161174. doi: 10.1371/journal.pone.0161174 (PMC4986962; doi:10.1371/journal.pone.0161174)
Supplement: S1 Appendix — (DOCX) [file pone.0161174.s001.docx]

**Supporting information:** The list of excluded articles.

1. Collins DC. Pseudomembranous enterocolitis. Further observations on the value of donor fecal enemata as an adjunct in the treatment of pseudomembranous enterocolitis. Am J Proctol. 1960;2:389-91. pmid: 13694740.

2. Schwan A, Sjölin S, Trottestam U, Aronsson B. Relapsing clostridium difficile enterocolitis cured by rectal infusion of homologous faeces. Lancet. 1983;2(8354):845.

3. Schwan A, Sjolin S, Trottestam U, Aronsson B. Relapsing Clostridium difficile enterocolitis cured by rectal infusion of normal faeces. Scand J Infect Dis. 1984;16(2):211-5. pmid: 6740251.

4. Bennet JD, Brinkman M. Treatment of ulcerative colitis by implantation of normal colonic flora. Lancet. 1989;1(8630):164.

5. Seal DV, Borriello SP, Piper M. Bacteriotherapy for clostridium difficile diarrhoea. Lancet. 1989;2(8662):558.

6. Tvede M, Rask-Madsen J. Bacteriotherapy for chronic relapsing Clostridium difficile diarrhoea in six patients. Lancet. 1989;1(8648):1156-60. pmid: 2566734.

7. Tvede M, Rask-Madsen J. Bacteriotherapy for Clostridium difficile diarrhoea. Lancet. 1990;335(8681):110. pmid: 1967389.

8. Shornikova AV, Casas IA, Mykkanen H, Salo E, Vesikari T. Bacteriotherapy with Lactobacillus reuteri in rotavirus gastroenteritis. Pediatr Infect Dis J. 1997;16(12):1103-7. pmid: 9427453.

9. Gionchetti P, Rizzello F, Venturi A, Brigidi P, Matteuzzi D, Bazzocchi G, et al. Oral bacteriotherapy as maintenance treatment in patients with chronic pouchitis: A double-blind, placebo-controlled trial. Gastroenterology 2000 119:2 (305-309). 2000;119(2):305-9.

10. Famularo G, Trinchieri V, De Simone C. Fecal bacteriotherapy or probiotics for the treatment of intestinal diseases? Am J Gastroenterol. 2001;96(7):2262-4. pmid: 11467668 doi: 10.1111/j.1572-0241.2001.03976.x.

11. Huovinen P. Bacteriotherapy: The time has come. British Medical Journal. 2001;323(7309):353-4.

12. Nye KJ, Turner T, Coleman DJ, Fallon D, Gee B, Messer S, et al. A comparison of the isolation rates of Salmonella and thermophilic Campylobacter species after direct inoculation of media with a dilute faecal suspension and undiluted faecal material. J Med Microbiol. 2001;50(8):659-62. pmid: 11478667 doi: 10.1099/0022-1317-50-8-659.

13. Bazzocchi G, Gionchetti P, Almerigi PF, Amadini C, Campieri M. Intestinal microflora and oral bacteriotherapy in irritable bowel syndrome. Dig Liver Dis. 2002;34(SUPPL. 2):S48-S53.

14. Borody TJ, Warren EF, Leis S, Surace R, Ashman O. Treatment of ulcerative colitis using fecal bacteriotherapy. J Clin Gastroenterol. 2003;37(1):42-7.

15. Brigidi P, Swennen E, Vitali B, Rossi M, Matteuzzi D. PCR detection of Bifidobacterium strains and Streptococcus thermophilus in feces of human subjects after oral bacteriotherapy and yogurt consumption. Int J Food Microbiol. 2003;81(3):203-9.

16. Borody TJ, Warren EF, Leis SM, Surace R, Ashman O, Siarakas S. Bacteriotherapy using fecal flora: Toying with human motions. J Clin Gastroenterol. 2004;38(6):475-83.

17. Gueimonde M, Sakata S, Kalliomaki M, Isolauri E, Benno Y, Salminen S. Effect of maternal consumption of lactobacillus GG on transfer and establishment of fecal bifidobacterial microbiota in neonates. J Pediatr Gastroenterol Nutr. 2006;42(2):166-70. pmid: 16456409 doi: 10.1097/01.mpg.0000189346.25172.fd.

18. Mueller MH, Geis M, Glatzle J, Kasparek M, Meile T, Jehle EC, et al. Risk of fecal diversion in complicated perianal Crohn's disease. J Gastrointest Surg. 2007;11(4):529-37. pmid: 17436140 doi: 10.1007/s11605-006-0029-3.

19. Pang X, Hua X, Yang Q, Ding D, Che C, Cui L, et al. Inter-species transplantation of gut microbiota from human to pigs. Isme j. 2007;1(2):156-62. pmid: 18043625 doi: 10.1038/ismej.2007.23.

20. Lepage P, Colombet J, Marteau P, Sime-Ngando T, Doré J, Leclerc M. Dysbiosis in inflammatory bowel disease: A role for bacteriophages? Gut. 2008;57(3):424-5.

21. You DM, Franzos MA, Holman RP. Successful treatment of fulminant Clostridium difficile infection with fecal bacteriotherapy. Ann Intern Med. 2008;148(8):632-3.

22. Bakken JS. Fecal bacteriotherapy for recurrent Clostridium difficile infection. Anaerobe. 2009;15(6):285-9.

23. Hellemans R, Naegels S, Holvoet J. Fecal transplantation for recurrent Clostridium difficile colitis, an underused treatment modality. Acta Gastroenterol Belg. 2009;72(2):269-70.

24. Johnson S. Recurrent Clostridium difficile infection: a review of risk factors, treatments, and outcomes. J Infect. 2009;58(6):403-10. pmid: 19394704 doi: 10.1016/j.jinf.2009.03.010.

25. Rubin TA, Gessert CE, Aas J. Stool transplantation for older patients with clostridium difficile infection. J Am Geriatr Soc. 2009;57(12):2386-7.

26. van Nood E, Speelman P, Kuijper EJ, Keller JJ. Struggling with recurrent Clostridium difficile infections: is donor faeces the solution? Euro Surveill. 2009;14(34). pmid: 19712646.

27. Arkkila PE, Uusitalo-Seppälä R, Lehtola L, Moilanen V, Ristikankare M, Mattila EJ. Fecal bacteriotherapy for recurrent clostridium difficile infection. Gastroenterology. 2010;138(5):S5.

28. Floch MH. Fecal bacteriotherapy, fecal transplant, and the microbiome. J Clin Gastroenterol. 2010;44(8):529-30. pmid: 20601895 doi: 10.1097/MCG.0b013e3181e1d6e2.

29. Grehan MJ, Borody TJ, Leis SM, Campbell J, Mitchell H, Wettstein A. Durable alteration of the colonic microbiota by the administration of donor fecal flora. J Clin Gastroenterol. 2010;44(8):551-61.

30. Kelly C, De Leon L. Successful treatment of recurrent clostridium difficile infection with donor stool administered at colonoscopy: A case series. Am J Gastroenterol. 2010;105:S135.

31. Kelly C, De Leon L, Kerstetter D, Okpara N. Barriers to greater utilization of fecal bacteriotherapy for chronic clostridium difficile infection. Am J Gastroenterol. 2010;105:S135-S6.

32. Manichanh C, Reeder J, Gibert P, Varela E, Llopis M, Antolin M, et al. Reshaping the gut microbiome with bacterial transplantation and antibiotic intake. Genome Res. 2010;20(10):1411-9. pmid: 20736229 PubMed Central PMCID: PMCPmc2945190 doi: 10.1101/gr.107987.110.

33. Miller CB, Dellon E, Isaacs K, Gangarosa L. Fecal bacteriotherapy via colonoscopy as rescue therapy for refractory and recurrent clostridium difficile - Associated diarrhea. Am J Gastroenterol. 2010;105:S323.

34. Rohlke F, Surawicz CM, Stollman N. Fecal Flora Reconstitution for recurrent clostridium difficile infection: Results and methodology. J Clin Gastroenterol. 2010;44(8):567-70.

35. Russell G, Kaplan J, Ferraro M, Michelow IC. Fecal bacteriotherapy for relapsing Clostridium difficile infection in a child: A proposed treatment protocol. Pediatrics. 2010;126(1):e239-e42.

36. Yoon SS, Brandt LJ. Treatment of refractory/recurrent C. difficile-associated disease by donated stool transplanted via colonoscopy: a case series of 12 patients. J Clin Gastroenterol. 2010;44(8):562-6. pmid: 20463588 doi: 10.1097/MCG.0b013e3181dac035.

37. Novel therapy for C. difficile infections. Infusions of donated feces may help those with recurrent infections. Harv Health Lett. 2011;36(12):7. pmid: 22121560.

38. Bakken JS, Borody T, Brandt LJ, Brill JV, Demarco DC, Franzos MA, et al. Treating Clostridium difficile infection with fecal microbiota transplantation. Clin Gastroenterol Hepatol. 2011;9(12):1044-9. pmid: 21871249 doi: 10.1016/j.cgh.2011.08.014.

39. Borody T, Campbell J, Torres M, Nowak A, Leis S. Reversal of idiopathic thrombocytopenic purpura [ITP] with fecal microbiota transplantation [FMT]. Am J Gastroenterol. 2011;106:S352.

40. Borody T, Leis S, Campbell J, Torres M, Nowak A. Fecal microbiota transplantation (FMT) in multiple sclerosis (MS). Am J Gastroenterol. 2011;106:S352.

41. Borody T, Torres M, Campbell J, Leis S, Nowak A. Reversal of inflammatory bowel disease (IBD) with recurrent faecal microbiota transplants (FMT). Am J Gastroenterol. 2011;106:S366.

42. Borody TJ, Campbell J. Fecal microbiota transplantation: current status and future directions. Expert Rev Gastroenterol Hepatol. 2011;5(6):653-5. pmid: 22017691 doi: 10.1586/egh.11.71.

43. Brandt LJ, Borody TJ, Campbell J. Endoscopic fecal microbiota transplantation: "First-line" treatment for severe clostridium difficile infection? J Clin Gastroenterol. 2011;45(8):655-7.

44. Girotra M, Bartlett J, Koerner K, Dutta S. Combined jejunal and colonic fecal bacteriotherapy in patients with recurrent clostridium difficile infection (RCDI). Am J Gastroenterol. 2011;106:S162-S3.

45. Glauser W. Risk and rewards of fecal transplants. Cmaj. 2011;183(5):541-2. pmid: 21398226 doi: 10.1503/cmaj.109-3806.

46. Gough E, Shaikh H, Manges AR. Systematic review of intestinal microbiota transplantation (fecal bacteriotherapy) for recurrent clostridium difficile infection. Clinical Infectious Diseases. 2011;53(10):994-1002.

47. Heuer H, Heuer L. Natural medicine: Fecal bacteriotherapy. Dtsch Apoth Ztg. 2011;151(24):58-9.

48. Ho N, Prasad V. Clostridium difficile diarrhea and fecal transplantation. J Clin Gastroenterol. 2011;45(8):742-3. pmid: 21555950 doi: 10.1097/MCG.0b013e31821b1094.

49. Ho N, Prasad V. Lacking the incentive to cure? Recurring Clostridium difficile diarrhea and our reluctance to use fecal transplantation. J Clin Gastroenterol. 2011;45(4):379-80. pmid: 21278582 doi: 10.1097/MCG.0b013e318202ad8e.

50. Kahn SA, Gorawara-Bhat R, Rubin DT. Fecal bacteriotherapy for ulcerative colitis; patients are ready, are we? Gastroenterology. 2011;140(5):S593.

51. Khoruts A, Sadowsky MJ. Therapeutic transplantation of the distal gut microbiota. Mucosal Immunol. 2011;4(1):4-7. pmid: 21150894 doi: 10.1038/mi.2010.79.

52. Landy J, Al-Hassi HO, McLaughlin SD, Walker AW, Ciclitira PJ, Nicholls RJ, et al. Review article: faecal transplantation therapy for gastrointestinal disease. Aliment Pharmacol Ther. 2011;34(4):409-15. pmid: 21682755 doi: 10.1111/j.1365-2036.2011.04737.x.

53. Mardani M. Intestinal microbiota transplantation for recurrent Clostridium difficile infection. Iranian Journal of Clinical Infectious Diseases. 2011;6(3):103.

54. Martin L. Modified fecal transplantation. J Clin Gastroenterol. 2011;45(8):742. pmid: 21555954 doi: 10.1097/MCG.0b013e3182166c8b.

55. Mellow M, Kanatzar A, Brandt L, Aroniadis O, Kelly C, Park T, et al. Longterm follow-up of colonoscopic Fecal Microbiota Transplant (FMT) for recurrent C. difficile infection (RCDI). Am J Gastroenterol. 2011;106:S149-S50.

56. Myers F. Beyond mainstream: making the case for fecal bacteriotherapy. Nursing. 2011;41(12):50-3.

57. Sofi A, Nawras A, Sodeman T, Garborg K, Silverman A. Fecal bacteriotherapy works for clostridium difficile infection - A meta-analysis. Am J Gastroenterol. 2011;106:S161.

58. Vignaroli C, Zandri G, Aquilanti L, Pasquaroli S, Biavasco F. Multidrug-resistant enterococci in animal meat and faeces and co-transfer of resistance from an Enterococcus durans to a human Enterococcus faecium. Curr Microbiol. 2011;62(5):1438-47. pmid: 21286720 doi: 10.1007/s00284-011-9880-x.

59. Wilcox GM. Early experience with a Fecal Bacteriotherapy (FB) program for recurrent and c-difficile infection (CDI). Gastroenterology. 2011;140(5):S361.

60. You D, Johnson M, Duplessis C, Speziale A. Successful Use of Fecal Bacteriotherapy in Severe Crohn's Colitis and Refractory Clostridium difficile Infection. Am J Gastroenterol. 2011;106:S315.

61. Fecal microbiota transplantation for treating recurrent Clostridium difficile infection. OR Manager. 2012;28(8):15-8. pmid: 22937601.

62. Anderson JL, Edney RJ, Whelan K. Systematic review: faecal microbiota transplantation in the management of inflammatory bowel disease. Aliment Pharmacol Ther. 2012;36(6):503-16. pmid: 22827693 doi: 10.1111/j.1365-2036.2012.05220.x.

63. Angelberger S, Lichtenberger C, Gratzer C, Papay P, Primas C, Eser A, et al. Fecal transplantation in patients with moderately to severely chronic active ulcerative colitis (UC). J Crohns Colitis. 2012;6:S159.

64. Bercik P, Lu J, Verdu EF, Deng Y, Wang C, Surette MG, et al. Transfer of IBS phenotype to GERM-free mice through microbiota transplantation. Neurogastroenterol Motil. 2012;24:33.

65. Borody T, Wettstein A, Campbell J, Leis S, Torres M, Finlayson S, et al. Fecal microbiota transplantation in ulcerative colitis: Review of 24 years experience. Am J Gastroenterol. 2012;107:S665.

66. Borody TJ, Campbell J. Fecal microbiota transplantation: techniques, applications, and issues. Gastroenterol Clin North Am. 2012;41(4):781-803. pmid: 23101687 doi: 10.1016/j.gtc.2012.08.008.

67. Borody TJ, Khoruts A. Fecal microbiota transplantation and emerging applications. Nat Rev Gastroenterol Hepatol. 2012;9(2):88-96. pmid: 22183182 doi: 10.1038/nrgastro.2011.244.

68. Brandt L, Aroniadis O. Long-term follow-up study of fecal microbiota transplantation (FMT) for ulcerative colitis (UC). Am J Gastroenterol. 2012;107:S657.

69. Brandt LJ. Editorial commentary: Fecal microbiota transplantation: Patient and physician attitudes. Clinical Infectious Diseases. 2012;55(12):1659-60.

70. Brandt LJ. Fecal transplantation for the treatment of Clostridium difficile infection. Gastroenterology and Hepatology. 2012;8(3):191-4.

71. Damman CJ, Miller SI, Surawicz CM, Zisman TL. The microbiome and inflammatory bowel disease: is there a therapeutic role for fecal microbiota transplantation? Am J Gastroenterol. 2012;107(10):1452-9. pmid: 23034604 doi: 10.1038/ajg.2012.93.

72. Duplessis CA, You D, Johnson M, Speziale A. Efficacious outcome employing fecal bacteriotherapy in severe Crohn's colitis complicated by refractory Clostridium difficile infection. Infection. 2012;40(4):469-72.

73. El-Matary W, Simpson R, Ricketts-Burns N. Fecal microbiota transplantation: are we opening a can of worms? Gastroenterology. 2012;143(2):e19; author reply e-20. pmid: 22732575 doi: 10.1053/j.gastro.2012.04.055.

74. Ferrecchia CE, Hobbs TR. A technique for orally administered fecal bacteriotherapy to treat chronic diarrhea in rhesus macaques (macaca mulatta). J Am Assoc Lab Anim Sci. 2012;51(5):655.

75. Floch MH. The power of poop: Probiotics and fecal microbial transplant. J Clin Gastroenterol. 2012;46(8):625-6.

76. Gallegos-Orozco JF, Paskvan-Gawryletz CD, Gurudu SR, Orenstein R. Successful colonoscopic fecal transplant for severe acute Clostridium difficile pseudomembranous colitis. Rev Gastroenterol Mex. 2012;77(1):40-2. pmid: 22450020.

77. Garg S, Walia R, Girotra M, Gjikopulli A, Mirza Y, Cuffari C, et al. A novel treatment for recurrent clostridium difficile infection in a 20-month-old. Am J Gastroenterol. 2012;107:S556.

78. Girotra M, Dutta A, Koerner K, Bodner B, Dutta SK. Recurrent clostridium difficile infection (RCDI) in geriatric patients: A long-term follow up of simultaneous jejunal and colonic administration of fecal bacteriotherapy (FT). Gastroenterology. 2012;142(5):S130.

79. Guo B, Harstall C, Louie T, Veldhuyzen Van Zanten S, Dieleman LA. Systematic review: Faecal transplantation for the treatment of Clostridium difficile-associated disease. Aliment Pharmacol Ther. 2012;35(8):865-75.

80. Jorup-Ronstrom C, Hakanson A, Sandell S, Edvinsson O, Midtvedt T, Persson AK, et al. Fecal transplant against relapsing Clostridium difficile-associated diarrhea in 32 patients. Scand J Gastroenterol. 2012;47(5):548-52. pmid: 22468996 doi: 10.3109/00365521.2012.672587.

81. Kahn SA, Gorawara-Bhat R, Rubin DT. Fecal bacteriotherapy for ulcerative colitis: Patients are ready, are we? Inflamm Bowel Dis. 2012;18(4):676-84.

82. Kahn SA, Vachon AI, Marks JA, Surma BL, Rodriquez DM, Goeppinger SR, et al. Patient perceptions of fecal microbiota transplantation for ulcerative colitis. Gastroenterology. 2012;142(5):S358.

83. Kahn SA, Young S, Rubin DT. Colonoscopic fecal microbiota transplant for recurrent clostridium difficile infection in a child. Am J Gastroenterol. 2012;107(12):1930-1.

84. Kassam Z, Hundal R, Marshall JK, Lee CH. Fecal transplant via retention enema for refractory or recurrent Clostridium difficile infection. Arch Intern Med. 2012;172(2):191-3. pmid: 22271132 doi: 10.1001/archinte.172.2.191.

85. Kelly CR, De Leon L, Jasutkar N. Fecal microbiota transplantation for relapsing clostridium difficile infection in 26 patients: Methodology and results. J Clin Gastroenterol. 2012;46(2):145-9.

86. Kruis W. Specific probiotics or 'fecal transplantation'. Digestive Diseases. 2012;30(SUPPL. 3):81-4.

87. Kunde S, Cloney D, Conrad H, Kugathasan S. Fecal microbial transplantation shows efficacy in children with refractory ulcerative colitis-early results of phase i clinical trial. Inflamm Bowel Dis. 2012;18:S66-S7.

88. Landy J, Perry-woodford ZL, Clark SK, Hart A. Patients' perspectives of faecal transplantation for pouchitis. J Crohns Colitis. 2012;6:S143.

89. Le Roy T, Llopis M, Bruneau A, Rabot S, Bevilacqua C, Martin P, et al. Gut microbiota transplantation demonstrates its causal role in the development of type 2 diabetes and fatty liver. Obesity Facts. 2012;5:41.

90. Lemon KP, Armitage GC, Relman DA, Fischbach MA. Microbiota-targeted therapies: an ecological perspective. Sci Transl Med. 2012;4(137):137rv5. pmid: 22674555 doi: 10.1126/scitranslmed.3004183.

91. Neelakanta A, Moudgal V, Upadhyay N, Valenstein P, Gunaratnam NT. Title: Successful treatment of refractory clostridium difficile infection(CDI) with intestinal microbiota transplant (IMT) in two patients with inflammatory bowel disease (IBD) and its effects on IBD. Gastroenterology. 2012;142(5):S395.

92. Neemann K, Eichele DD, Smith PW, Bociek R, Akhtari M, Freifeld A. Fecal microbiota transplantation for fulminant Clostridium difficile infection in an allogeneic stem cell transplant patient. Transpl Infect Dis. 2012;14(6):E161-5. pmid: 23121625 doi: 10.1111/tid.12017.

93. Postigo R, Kim JH. Colonoscopic versus nasogastric fecal transplantation for the treatment of Clostridium difficile infection: a review and pooled analysis. Infection. 2012;40(6):643-8. pmid: 22847629 doi: 10.1007/s15010-012-0307-9.

94. Rohlke F, Stollman N. Fecal microbiota transplantation in relapsing Clostridium difficile infection. Therap Adv Gastroenterol. 2012;5(6):403-20.

95. Shahinas D, Silverman M, Sittler T, Chiu C, Kim P, Allen-Vercoe E, et al. Toward an understanding of changes in diversity associated with fecal microbiome transplantation based on 16S rRNA gene deep sequencing. MBio. 2012;3(5). pmid: 23093385 doi: 10.1128/mBio.00338-12.

96. Singh N, Suskind D, Wahbeh G. Fecal bacteriotherapy in a 6 year old patient with ulcerative colitis and clostridium difficile. Inflamm Bowel Dis. 2012;18:S69.

97. Stollman N, Surawicz C. Fecal transplant for Clostridium difficile. Arch Intern Med. 2012;172(10):825.

98. Vrieze A, Van Nood E, Holleman F, Salojarvi J, Kootte RS, Bartelsman JF, et al. Transfer of intestinal microbiota from lean donors increases insulin sensitivity in individuals with metabolic syndrome. Gastroenterology. 2012;143(4):913-6.e7. pmid: 22728514 doi: 10.1053/j.gastro.2012.06.031.

99. Watson JB, Habr F, Kelly C. First reported complication of fecal microbiota transplant: Ulcerative colitis flare after FMT for relapsing clostridium difficile infection. Gastroenterology. 2012;142(5):S540.

100. Weissman JS, Coyle W. Stool transplants: ready for prime time? Curr Gastroenterol Rep. 2012;14(4):313-6. pmid: 22585070 doi: 10.1007/s11894-012-0263-7.

101. Zhang F, Luo W, Shi Y, Fan Z, Ji G. Should we standardize the 1,700-year-old fecal microbiota transplantation. Am J Gastroenterol. 2012;107(11):1755.

102. Zipursky JS, Sidorsky TI, Freedman CA, Sidorsky MN, Kirkland KB. Patient attitudes toward the use of fecal microbiota transplantation in the treatment of recurrent Clostridium difficile infection. Clin Infect Dis. 2012;55(12):1652-8. pmid: 22990849 doi: 10.1093/cid/cis809.

103. ALS Untangled No. 21: Fecal transplants. Amyotroph Lateral Scler Frontotemporal Degener. 2013;14(5-6):482-5. pmid: 23870000 doi: 10.3109/21678421.2013.814981.

104. Fecal microbiota transplantation for treatment of recurrent C. difficile infection. Clin Privil White Pap. 2013;(246):1-15. pmid: 24133730.

105. Fecal microbiota transplantation for treating recurrent Clostridium difficile infection. Manag Care. 2013;22(6):18-9. pmid: 23841136.

106. Donor faeces for recurrent Clostridium difficile diarrhoea? Bmj. 2013;346:f376. pmid: 23344312 doi: 10.1136/bmj.f376.

107. Adamu BO, Lawley TD. Bacteriotherapy for the treatment of intestinal dysbiosis caused by Clostridium difficile infection. Current Opinion in Microbiology. 2013;16(5):596-601.

108. Agito MD, Atreja A, Rizk MK. Fecal microbiota transplantation for recurrent C difficile infection: ready for prime time? Cleve Clin J Med. 2013;80(2):101-8. pmid: 23376915 doi: 10.3949/ccjm.80a.12110.

109. Allen-Vercoe E, Petrof EO. Artificial stool transplantation: progress towards a safer, more effective and acceptable alternative. Expert Rev Gastroenterol Hepatol. 2013;7(4):291-3. pmid: 23639085 doi: 10.1586/egh.13.16.

110. Alsakka M, Sharabash N, Alktaifi A, Salih M, German M. Successful fecal microbiota transplantation (FMT) for recurrent clostridium difficile infection (CDI) after subtotal colectomy. Am J Gastroenterol. 2013;108:S365-S6.

111. Aroniadis OC, Brandt LJ. Fecal microbiota transplantation: past, present and future. Curr Opin Gastroenterol. 2013;29(1):79-84. pmid: 23041678 doi: 10.1097/MOG.0b013e32835a4b3e.

112. Aroniadis OC, Brandt LJ, Greenberg A, Borody TJ, Kelly C, Mellow M, et al. Long-term follow-up study of fecal microbiota transplantation (FMT) for severe or complicated clostridium difficile infection (CDI). Gastroenterology. 2013;144(5):S185.

113. Avery L, Hasan M. Fecal bacteriotherapy for clostridium difficile infections - its time has come. Clin. Microbiol. Newsl. 2013;35(15):119-24.

114. Balzola F, Cullen G, Hoentjen F, Ho GT, Russell R. Safety, tolerability, and clinical response after fecal transplantation in children and young adults with ulcerative colitis. Inflammatory Bowel Disease Monitor. 2013;13(4):167.

115. Bansal S, Serban R, Kemal N, Casey K, Dunnigan K, Kurchin A. Fecal microbiota transplant for recurrent clostridium difficile infection at a teaching hospital in upstate New York: Our experience. Am J Gastroenterol. 2013;108:S383-S4.

116. Barbut F. Alleviating the burden of CDI: Current and emerging treatment options. Int J Antimicrob Agents.2013;42:S11.

117. Baron TH, Kozarek RA. Fecal microbiota transplant: we know its history, but can we predict its future? Mayo Clin Proc. 2013;88(8):782-5. pmid: 23910405 doi: 10.1016/j.mayocp.2013.06.007.

118. Baty V, Mougin B. What about public perception of antibiotics in the era of the fecal microbiota transplantation? between the devil and the deep blue sea. Am J Gastroenterol. 2013;108(9):1540.

119. Berg AM, Farraye FA. Duodenal infusion of stool is more effective than vancomycin in patients with recurrent Clostridium difficile. Evid Based Med. 2013;18(6):220-1. pmid: 23616152 doi: 10.1136/eb-2013-101308.

120. Blosser R. Probiotic infusion during colonoscopy is an effective therapeutic alternative for refractory or recurrent C. Difficile colitis. Am J Gastroenterol. 2013;108:S181.

121. Bookstaver PB, Ahmed Y, Millisor VE, Siddiqui W, Albrecht H. Clostridium difficile: case report and concise review of fecal microbiota transplantation. J S C Med Assoc. 2013;109(2):62-6. pmid: 24902394.

122. Borody TJ, Brandt LJ, Paramsothy S, Agrawal G. Fecal microbiota transplantation: a new standard treatment option for Clostridium difficile infection. Expert Rev Anti Infect Ther. 2013;11(5):447-9. pmid: 23627849 doi: 10.1586/eri.13.26.

123. Borody TJ, Paramsothy S, Agrawal G. Fecal microbiota transplantation: indications, methods, evidence, and future directions. Curr Gastroenterol Rep. 2013;15(8):337. pmid: 23852569 PubMed Central PMCID: PMCPmc3742951 doi: 10.1007/s11894-013-0337-1.

124. Borody TJ, Peattie D, Campbell J. Therapeutic potential of the human gastrointestinal microbiome. Drug Development Research. 2013;74(6):385-92.

125. Brandt LJ. American Journal of Gastroenterology Lecture: Intestinal microbiota and the role of fecal microbiota transplant (FMT) in treatment of C. difficile infection. Am J Gastroenterol. 2013;108(2):177-85. pmid: 23318479 doi: 10.1038/ajg.2012.450.

126. Brandt LJ, Aroniadis OC. An overview of fecal microbiota transplantation: techniques, indications, and outcomes. Gastrointest Endosc. 2013;78(2):240-9. pmid: 23642791 doi: 10.1016/j.gie.2013.03.1329.

127. Broecker F, Kube M, Klumpp J, Schuppler M, Biedermann L, Hecht J, et al. Analysis of the intestinal microbiome of a recovered Clostridium difficile patient after fecal transplantation. Digestion. 2013;88(4):243-51. pmid: 24335204 doi: 10.1159/000355955.

128. Burke KE, Lamont JT. Fecal transplantation for recurrent Clostridium difficile infection in older adults: a review. J Am Geriatr Soc. 2013;61(8):1394-8. pmid: 23869970 doi: 10.1111/jgs.12378.

129. Callewaert C, Plaquet T, Bostoen J, Van De Wiele T, Boon N. Axillary bacterial transplantation and bacteriotherapy as a promising technique to treat bromhidrosis. J Invest Dermatol. 2013;133:S207.

130. Chan YK, Estaki M, Gibson DL. Clinical consequences of diet-induced dysbiosis. Ann Nutr Metab. 2013;63(SUPPL.2):28-40.

131. Claud EC, Keegan KP, Brulc JM, Lu L, Bartels D, Glass E, et al. Bacterial community structure and functional contributions to emergence of health or necrotizing enterocolitis in preterm infants. Microbiome. 2013;1(1).

132. Collins SM, Kassam Z, Bercik P. The adoptive transfer of behavioral phenotype via the intestinal microbiota: Experimental evidence and clinical implications. Curr Opin Microbiol.2013;16(3):240-5.

133. Crouzet L, Gaultier E, Del'Homme C, Cartier C, Delmas E, Dapoigny M, et al. The hypersensitivity to colonic distension of IBS patients can be transferred to rats through their fecal microbiota. Neurogastroenterol Motil. 2013;25(4):e272-82. pmid: 23433203 doi: 10.1111/nmo.12103.

134. Davidovics ZH, Hyams JS. Fecal transplantation: re-discovering the value of stool. Curr Opin Pediatr. 2013;25(5):618-23. pmid: 23995428 doi: 10.1097/MOP.0b013e328363ed66.

135. Dinan TG, Cryan JF. Melancholic microbes: A link between gut microbiota and depression? Neurogastroenterol Motil. 2013;25(9):713-9.

136. El-Matary W. Fecal microbiota transplantation: Long-term safety issues. Am J Gastroenterol. 2013;108(9):1537-8.

137. Ettinger G, Burton JP, Reid G. If microbial ecosystem therapy can change your life, what's the problem? BioEssays. 2013;35(6):508-12.

138. Ferrecchia CE, Hobbs TR. Efficacy of oral fecal bacteriotherapy in rhesus macaques (Macaca mulatta) with chronic diarrhea. Comparative Medicine. 2013;63(1):71-5.

139. Fox JL. Fecal transplants to follow FDA rules. Nat Biotechnol. 2013;31(7):583. pmid: 23839135 doi: 10.1038/nbt0713-583.

140. Ganc A, Ganc R, Frisoli Jr A, Pasternak J. Fecal transplantation-an original per-oral endoscopic technique with a pediatric colonoscope. J Gastroenterol Hepatol. 2013;28:115.

141. Garg S, Fricke WF, Girotra M, Dutta A, Von Rosenvinge EC, Dutta S. Recurrent clostridium difficile infection: A longitudinal study of alterations in fecal microbiome in patients-donor pairs before and after fecal microbiota therapy. Gastroenterology. 2013;144(5):S184-S5.

142. Garg S, Fricke WF, Girotra M, Von Rosenvinge EC, Dutta A, Dutta SK. Emerging role of fecal microbiota therapy in the treatment of recurrent clostridium difficile infection in children. Gastroenterology. 2013;144(5):S45.

143. Gens KD, Elshaboury RH, Holt JS. Fecal microbiota transplantation and emerging treatments for Clostridium difficile infection. J Pharm Pract. 2013;26(5):498-505.

144. Graham D, Attumi T, Opekun A, Metcalf G, Muzny D, Hyde E, et al. Triple bacteroides fecal replacement therapy for relapsing clostridium difficile diarrhea (fecal transplantation sans feces). Am J Gastroenterol. 2013;108:S170.

145. Gutman J, Kurchin A. Split donation fecal microbiota transplantation. Am J Gastroenterol. 2013;108(10):1659-60.

146. Hamilton MJ, Weingarden AR, Unno T, Khoruts A, Sadowsky MJ. High-throughput DNA sequence analysis reveals stable engraftment of gut microbiota following transplantation of previously frozen fecal bacteria. Gut Microbes. 2013;4(2):125-35. pmid: 23333862 doi: 10.4161/gmic.23571.

147. Hataye JM, Palmore TN, Powers Iii JH. Duodenal infusion of feces for recurrent Clostridium difficile [2]. N Engl J Med. 2013;368(22):2143-4.

148. Hodes RM. Fecal flora reconstitution for amyotrophic lateral sclerosis. J Clin Gastroenterol. 2013;47(7):655. pmid: 23507766 doi: 10.1097/MCG.0b013e3182897a14.

149. Ihunnah C, Khoruts A, Fischer M, Afzali A, Aroniadis O, Barto A, et al. Fecal microbiota transplantation (FMT) for treatment of clostridium difficile infection (CDI) in immunocompromised patients ACG governors award for excellence in clinical research. Am J Gastroenterol. 2013;108:S179-S80.

150. Jiang ZD, Hoang LN, Lasco TM, Garey KW, DuPont HL. Physician attitudes toward the use of fecal transplantation for recurrent Clostridium difficile infection in a metropolitan area. Clinical Infectious Diseases. 2013;56(7):1059-60.

151. Jump RLP. Clostridium difficile infection in older adults. Aging Health. 2013;9(4):403-14.

152. Kahn SA, Vachon A, Rodriquez D, Goeppinger SR, Surma B, Marks J, et al. Patient perceptions of fecal microbiota transplantation for ulcerative colitis. Inflamm Bowel Dis. 2013;19(7):1506-13. pmid: 23624888 doi: 10.1097/MIB.0b013e318281f520.

153. Kao D, Madsen K. Fecal microbiota transplantation (FMT) in the treatment of inflammatory bowel disease (IBD): A case report acg/astrazeneca clinical vignette award. Am J Gastroenterol. 2013;108:S415-S6.

154. Karadsheh Z, Sule S. Fecal transplantation for the treatment of recurrent Clostridium difficile infection. N Am J Med Sci. 2013;5(6):339-43.

155. Kassam Z, Lee CH, Yuan Y, Hunt RH. Navigating long-term safety in fecal microbiota transplantation. Am J Gastroenterol. 2013;108(9):1538. pmid: 24005359 doi: 10.1038/ajg.2013.214.

156. Kassam Z, Lee CH, Yuan Y, Hunt RH. Fecal microbiota transplantation for Clostridium difficile infection: systematic review and meta-analysis. Am J Gastroenterol. 2013;108(4):500-8. pmid: 23511459 doi: 10.1038/ajg.2013.59.

157. Kelly CP. Fecal microbiota transplantation - An old therapy comes of age. N Engl J Med. 2013;368(5):474-5.

158. Khanna S, Kashyap P, Rainey J, Loftus E, Pardi D. Outcomes from fecal microbiota transplantation in adults with C. difficile infection and inflammatory bowel disease. Am J Gastroenterol. 2013;108:S508.

159. Kleger A, Schnell J, Essig A, Wagner M, Bommer M, Seufferlein T, et al. Fecal transplant in refractory Clostridium difficile colitis. Dtsch Arztebl Int. 2013;110(7):108-15. pmid: 23468820 doi: 10.3238/arztebl.2013.0108.

160. Knight CL, Surawicz CM. Clostridium difficile Infection. Med Clin North Am. 2013;97(4):523-36.

161. Koenigsknecht MJ, Young VB. Faecal microbiota transplantation for the treatment of recurrent Clostridium difficile infection: current promise and future needs. Curr Opin Gastroenterol. 2013;29(6):628-32. pmid: 24100717 doi: 10.1097/MOG.0b013e328365d326.

162. Konstantinov SR, Peppelenbosch MP. Fecal microbiota transfer may increase irritable bowel syndrome and inflammatory bowel diseases-associated bacteria. Gastroenterology. 2013;144(4):e19-20. pmid: 23462124 doi: 10.1053/j.gastro.2012.12.040.

163. Kump PK, Gröchenig HP, Lackner S, Trajanovski S, Reicht G, Hoffmann KM, et al. Successful improvement of dysbiosis by fecal microbiota transplantation is not sufficient to induce clinical remission in chronic active ulcerative colitis. J Crohns Colitis. 2013;7:S155.

164. Landy J, Al-Hassi HO, Mann ER, Peake ST, McLaughlin SD, Ciclitira PJ, et al. The effects of fecal microbiota transplantation on the innate immune system and epithelial tight junction expression in chronic refractory pouchitis. Gastroenterology. 2013;144(5):S301.

165. Landy J, Al-Hassi HO, Mann ER, Peake ST, McLaughlin SD, Ciclitira PJ, et al. A prospective controlled pilot study of fecal microbiota transplantation for chronic refractory pouchitis. Gastroenterology. 2013;144(5):S897.

166. Landy J, Omar Al-Hassi H, Ronde E, Mann E, Peake S, McLaughlin S, et al. The effects of faecal microbiota transplantation on the innate immune system and epithelial tight junction expression in chronic refractory pouchitis. J Crohns Colitis. 2013;7:S15.

167. Landy J, Omar Al-Hassi H, Ronde E, Mann E, Peake S, McLaughlin S, et al. A prospective controlled pilot study of faecal microbiota transplantation for chronic refractory pouchitis. J Crohns Colitis. 2013;7:S247-S8.

168. Lehrer S. Duodenal infusion of feces for recurrent Clostridium difficile [4]. N Engl J Med. 2013;368(22):2144.

169. Lewis SS, Anderson DJ. Treatment of Clostridium difficile infection: Recent trial results. Clinical Investigation. 2013;3(9):875-86.

170. Lofland D, Josephat F, Partin S. Fecal transplant for recurrent Clostridium difficile infection. Clin Lab Sci. 2013;26(3):131-5. pmid: 23967542.

171. Luna R, Pitashny M, Runge J, Shang Y, Hollister E, Nagy-Szakal D, et al. Microbiome characterization as a diagnostic tool in fecal microbiome transplantation. J Mol Diagn. 2013;15(6):874-5.

172. McKinney M. FDA slaps regs on fecal transplants. Increased steps for C. diff treatment draw mixed reactions from providers. Mod Healthc. 2013;43(21):10. pmid: 23947094.

173. McKinney M. Despite 'eww' factor... fecal transplants gain ground against C. diff. Mod Healthc. 2013;43(4):12-3. pmid: 23488112.

174. Miao J, Powell J, Martin S, Shah S, Fester K, Scheffert J, et al. Clostridium difficile following solid organ transplantation. Pharmacotherapy. 2013;33(10):e295.

175. Mikamo H. Treatment for Clostridium difficile infections. Int J Antimicrob Agents. 2013;42:S16.

176. Mitchell I, Shropshire K, Ruel J. Clostridium difficile infection and fecal bacteriotherapy. Gastroenterol Nurs. 2013;36(1):42-50.

177. Mole B. FDA gets to grips with faeces. Nature. 2013;498(7453):147-8. pmid: 23765468 doi: 10.1038/498147a.

178. Nagy GG, Várvölgyi C, Balogh Z, Orosi P, Paragh G. Detailed methodological recommendations for the treatment of Clostridium diffi cile-associated diarrhea with faecal transplantation. Orvosi Hetilap. 2013;154(1):10-9.

179. Oprita R, Kostov A, Musat F. Clostridium difficile-associated diarrhea, a new challenge. Eur J Intern Med. 2013;24:e73.

180. Orenstein R, Griesbach CL, Dibaise JK. Moving fecal microbiota transplantation into the mainstream. Nutr Clin Pract. 2013;28(5):589-98.

181. Owens C, Broussard E, Surawicz C. Fecal microbiota transplantation and donor standardization. Trends Microbiol. 2013;21(9):443-5.

182. Paasche S. Fecal microbiota transplantation: an innovative approach to treating Clostridium difficile disease. Jaapa. 2013;26(8):46-9. pmid: 24049940.

183. Patel NC, Griesbach CL, DiBaise JK, Orenstein R. Fecal microbiota transplant for recurrent Clostridium difficile infection: Mayo Clinic in Arizona experience. Mayo Clin Proc. 2013;88(8):799-805. pmid: 23910407 doi: 10.1016/j.mayocp.2013.04.022.

184. Patel S, Kelly C, Colombel JF, Atreja A. Comparative cost analysis of fecal microbiota transplant and antibiotic treatment for recurrent clostridium difficile infection. Am J Gastroenterol. 2013;108:S169-S70.

185. Petrof EO, Gloor GB, Vanner SJ, Weese SJ, Carter D, Daigneault MC, et al. Stool substitute transplant therapy for the eradication of Clostridium difficile infection: 'RePOOPulating' the gut. Microbiome. 2013;1(1).

186. Pinn D, Aroniadis O, Brandt L. Follow-up study of fecal microbiota transplantation (FMT) for the treatment of refractory irritable bowel syndrome (IBS). Am J Gastroenterol. 2013;108:S563.

187. Potakamuri L, Turnbough L, Maheshwari A, Kantsevoy S, Ofosu A, Thuluvath P, et al. Effectiveness of fecal microbiota transplantation for the treatment of recurrent clostridium difficile infection: Community hospital experience. Am J Gastroenterol. 2013;108:S175.

188. Ramsauer B. Duodenal infusion of feces for recurrent Clostridium difficile [3]. N Engl J Med. 2013;368(22):2144.

189. Rogers GB, Bruce KD. Challenges and opportunities for faecal microbiota transplantation therapy. Epidemiol Infect. 2013;141(11):2235-42. pmid: 23735045 doi: 10.1017/s0950268813001362.

190. Rubin TA, Gessert CE, Aas J, Bakken JS. Fecal microbiome transplantation for recurrent Clostridium difficile infection: report on a case series. Anaerobe. 2013;19:22-6. pmid: 23182843 doi: 10.1016/j.anaerobe.2012.11.004.

191. Sampath K, Levy LC, Gardner TB. Fecal transplantation: Beyond the aesthetic. Gastroenterology. 2013;145(5):1151-3.

192. Senior K. Faecal transplantation for recurrent C difficile diarrhoea. The Lancet infectious diseases. 2013;13(3):200-1.

193. Smith S. Intestinal microbiota transplantation: A case of Crohn's colitis with superimposed Clostridium difficile infection. West Indian Medical Journal. 2013;62(7):675-7.

194. Smits LP, Bouter KE, de Vos WM, Borody TJ, Nieuwdorp M. Therapeutic potential of fecal microbiota transplantation. Gastroenterology. 2013;145(5):946-53. pmid: 24018052 doi: 10.1053/j.gastro.2013.08.058.

195. Sofi A, Georgescu C, Sodeman T, Nawras A. Physician outlook towards fecal microbiota transplantation in the treatment of recurrent clostridium difficile infection. Gastroenterology. 2013;144(5):S241.

196. Sofi AA, Silverman AL, Khuder S, Garborg K, Westerink JMA, Nawras A. Relationship of symptom duration and fecal bacteriotherapy in Clostridium difficile infection-pooled data analysis and a systematic review. Scand J Gastroenterol. 2013;48(3):266-73.

197. Song Y, Garg S, Girotra M, Maddox C, von Rosenvinge EC, Dutta A, et al. Microbiota dynamics in patients treated with fecal microbiota transplantation for recurrent Clostridium difficile infection. PLoS One. 2013;8(11):e81330. pmid: 24303043 doi: 10.1371/journal.pone.0081330.

198. Soota K, Telfah M, Ramesh N, Pereira M, Lingutla D. Treatment of recurrent clostridium difficile infection with combined jejunal and colonic fecal microbiota transplant. Am J Gastroenterol. 2013;108:S398.

199. Suwantarat N, Bobak DA. Fecal bacteriotherapy for recurrent clostridium difficile infection: What's old is new again? Curr Infect Dis Rep. 2013;15(2):101-3.

200. Turner JD. Evaluation of clostridium difficile treatment with fecal microbiota transplantation. Am J Trop Med Hyg. 2013;89(5):217.

201. Van den Abbeele P, Verstraete W, El Aidy S, Geirnaert A, Van de Wiele T. Prebiotics, faecal transplants and microbial network units to stimulate biodiversity of the human gut microbiome. Microb Biotechnol. 2013;6(4):335-40. pmid: 23594389 doi: 10.1111/1751-7915.12049.

202. Van Schooneveld TC, Gross A, Kalil AC. Duodenal infusion of feces for recurrent Clostridium difficile [1]. N Engl J Med. 2013;368(22):2143.

203. Vaughn B, Kahn S, Rubin D, Moss A. Donor stool preparation for fecal transplantation in patients with IBD: Regulatory and financial aspects. Inflamm Bowel Dis. 2013;19:S88.

204. Vindigni SM, Broussard EK, Surawicz CM. Alteration of the intestinal microbiome: fecal microbiota transplant and probiotics for Clostridium difficile and beyond. Expert Rev Gastroenterol Hepatol. 2013;7(7):615-28. pmid: 24070153 doi: 10.1586/17474124.2013.832501.

205. Vrieze A, De Groot PF, Kootte RS, Knaapen M, Van Nood E, Nieuwdorp M. Fecal transplant: A safe and sustainable clinical therapy for restoring intestinal microbial balance in human disease? Best Pract Res Clin Gastroenterol. 2013;27(1):127-37.

206. Vyas D, L'Esperance HE, Vyas A. Stool therapy may become a preferred treatment of recurrent Clostridium difficile? World J Gastroenterol. 2013;19(29):4635-7.

207. Waite DW, Deines P, Taylor MW. Quantifying the impact of storage procedures for faecal bacteriotherapy in the critically endangered New Zealand Parrot, the Kakapo (Strigops habroptilus). Zoo Biol. 2013;32(6):620-5.

208. Weingarden A, Hamilton MJ, Sadowsky MJ, Khoruts A. Changes in bacterial composition following fecal microbiota transplantation for severe clostridium difficie infection. Gastroenterology. 2013;144(5):S829.

209. Weingarden AR, Hamilton MJ, Sadowsky MJ, Khoruts A. Resolution of severe clostridium difficile infection following sequential fecal microbiota transplantation. J Clin Gastroenterol. 2013;47(8):735-7.

210. Youssef MA, Gavin M. Fecal microbiota transplant: A case report in an immunosuppressed patient with crohn's disease and recurrent clostridium difficile infection. Gastroenterology. 2013;144(5):S626.

211. Zhang F, Wang H, Wang M, Cui B, Huang G, Ji G, et al. Standard fecal microbiota transplantation through mid-gut is an effective therapy of refractory Crohn's disease. J Gastroenterol Hepatol. 2013;28:9.

212. Faecal microbiota transplantation. Drug Ther Bull. 2014;52(12):141-4. pmid: 25505015 doi: 10.1136/dtb.2014.12.0298.

213. Solving a C.difficile problem. If antibiotics fail, a stool transplant can help cure a severe infection. Johns Hopkins Med Lett Health After 50. 2014;25(13):3. pmid: 24809121.

214. Agrawal M, Aroniadis OC, Brandt LJ, Kelly C, Freeman S, Surawicz C, et al. A long-term follow-up study of the efficacy and safety of fecal microbiota transplant (FMT) for Recurrent/Severe/Complicated C. Difficile Infection (CDI) in the elderly. Gastroenterology. 2014;146(5):S42-S3.

215. Alhmoud T, Gavin M. An unusual complication after a fecal microbiota transplant via colonoscopy. Am J Gastroenterol. 2014;109:S424.

216. Allegretti JR, Hamilton MJ. Restoring the gut microbiome for the treatment of inflammatory bowel diseases. World J Gastroenterol. 2014;20(13):3468-74. pmid: 24707129 doi: 10.3748/wjg.v20.i13.3468.

217. Allegretti JR, Korzenik JR, Hamilton MJ. Fecal microbiota transplantation via colonoscopy for recurrent C. difficile Infection. J Vis Exp. 2014;(94). pmid: 25549239 doi: 10.3791/52154.

218. Anand R, Girotra M, Garg S, Dutta S. Safety and efficacy of fecal microbiota transplantation (FMT) for recurrent clostridium difficile infection (RCDI) in septuagenarians, octogenarians, and nonegenarians: A single-center experience. Am J Gastroenterol. 2014;109:S195.

219. Arkkila PE, Mattila E, Kainulainen V, Satokari R. Simple and practical frozen preparation for transplantation of fecal microbiota for recurrent clostridium difficile infection. Gastroenterology. 2014;146(5):S193-S4.

220. Atkins KA, Kao D. Potential cost savings associated with timely fecal microbiota transplantation (FMT) for recurrent clostridium difficile infection (RCDI). Gastroenterology. 2014;146(5):S-252.

221. Austin M, Mellow M, Tierney WM. Fecal microbiota transplantation in the treatment of Clostridium difficile infections. Am J Med. 2014;127(6):479-83. pmid: 24582877 doi: 10.1016/j.amjmed.2014.02.017.

222. Axelrad J, Shah B. C. Difficile infection in inflammatory bowel disease: A nursing-based quality improvement strategy. Am J Gastroenterol. 2014;109:S639-S40.

223. Bakken JS. Staggered and tapered antibiotic withdrawal with administration of kefir for recurrent Clostridium difficile infection. Clinical Infectious Diseases. 2014;59(6):858-61.

224. Barzoloski-O'Connor B. Clostridium difficile: Transferring C. difficile spores causes an infection that leads to 14,000 deaths annually in the U.S. Nursing Critical Care. 2014;9(4):30-4.

225. Borody TJ, Brandt LJ, Paramsothy S. Therapeutic faecal microbiota transplantation: current status and future developments. Curr Opin Gastroenterol. 2014;30(1):97-105. pmid: 24257037 doi: 10.1097/mog.0000000000000027.

226. Borody TJ, Finlayson S. Fecal microbiota transplantation for Clostridium difficile infection: A surgeon's perspective. Seminars in Colon and Rectal Surgery. 2014;25(3):163-6.

227. Borody TJ, Finlayson S, Paramsothy S. Is Crohn's disease ready for fecal Microbiota transplantation? J Clin Gastroenterol. 2014;48(7):582-3.

228. Brace C, Gloor GB, Ropeleski M, Allen-Vercoe E, Petrof EO. Microbial composition analysis of Clostridium difficile infections in an ulcerative colitis patient treated with multiple fecal microbiota transplantations. J Crohns Colitis. 2014;8(9):1133-7.

229. Brown AC. Ulcerative colitis, Crohn's disease and irritable bowel syndrome patients need fecal transplant research and treatment. J Crohns Colitis. 2014;8(2):179.

230. Brown WR. Fecal microbiota transplantation in treating Clostridium difficile infection. J Dig Dis. 2014;15(8):405-8. pmid: 24825534 doi: 10.1111/1751-2980.12160.

231. Cammarota G, Ianiro G, Bibbo S, Gasbarrini A. Fecal microbiota transplantation: a new old kid on the block for the management of gut microbiota-related disease. J Clin Gastroenterol. 2014;48 Suppl 1:S80-4. pmid: 25291136 doi: 10.1097/mcg.0000000000000244.

232. Cammarota G, Ianiro G, Bibbo S, Gasbarrini A. Gut microbiota modulation: probiotics, antibiotics or fecal microbiota transplantation? Intern Emerg Med. 2014;9(4):365-73. pmid: 24664520 doi: 10.1007/s11739-014-1069-4.

233. Cammarota G, Ianiro G, Bibbò S, Gasbarrini A. Fecal microbiota transplantation a new old kid on the block for the management of gut microbiota-related disease. J Clin Gastroenterol. 2014;48:S80-S4.

234. Cammarota G, Ianiro G, Gasbarrini A. Fecal microbiota transplantation for the treatment of clostridium difficile infection: A systematic review. J Clin Gastroenterol. 2014;48(8):693-702.

235. Cammarota G, Ianiro G, Gasbarrini A, Masucci L, Sanguinetti M. Faecal transplantation for Clostridium difficile infection. Three cases treated in Italy. Digestive and Liver Disease. 2014;46(5):475.

236. Chen WX, Ren LH, Shi RH. Enteric microbiota leads to new therapeutic strategies for ulcerative colitis. World J Gastroenterol. 2014;20(42):15657-63.

237. Cherem JH, Ulloa IH. Home fecal transplantation in elderly women. Gac Med Mex. 2014;150(1):106-7.

238. Chetan M, Benjamin H, Ajin J, Alireza M, Nichole M, Mayur R. Fecal transplant for recurrent and/or refractory clostridium difficile infection in patients with inflammatory bowel disease. Inflamm Bowel Dis. 2014;20:S72.

239. Chuang P, Walsh E, Kim S, Lee T, Hwang JI, Cho I. Differential response to anticoagulation in germ-free swiss webster mice conventionalized with fecal transplantation from human donors requiring varying doses of warfarin. Gastroenterology. 2014;146(5):S-543.

240. Colleen K, Hassan Z, Stacy K. New diagnosis of crohn's colitis 6 weeks after fecal microbiota transplantation (FMT). Inflamm Bowel Dis. 2014;20:S21.

241. Colman RJ, Rubin DT. Fecal microbiota transplantation as therapy for inflammatory bowel disease: a systematic review and meta-analysis. J Crohns Colitis. 2014;8(12):1569-81. pmid: 25223604 doi: 10.1016/j.crohns.2014.08.006.

242. Costello S, La Brooy J, Tucker E, Holloway R, Schoeman M, Andrews JM. Establishment of a fecal microbiota transplant service for the treatment of recurrent Clostridium difficile colitis in the Australian public hospital setting. J Gastroenterol Hepatol (Australia). 2014;29:134.

243. Crabtree S, Gupta J. Knowledge and attitudes towards faecal bacteriotherapy on ITU. Intensive Care Med. 2014;40(1):S106.

244. D'Haens GR, Sartor RB, Silverberg MS, Petersson J, Rutgeerts P. Future directions in inflammatory bowel disease management. J Crohns Colitis. 2014;8(8):726-34.

245. Dai YX, Shi CB, Cui BT, Wang M, Ji GZ, Zhang FM. Fecal microbiota transplantation and prednisone for severe eosinophilic gastroenteritis. World J Gastroenterol. 2014;20(43):16368-71. pmid: 25473198 doi: 10.3748/wjg.v20.i43.16368.

246. Damman C, Brittnacher M, Hayden H, Radey M, Hager K, Miller S, et al. Single colonoscopically administered fecal microbiota transplant for ulcerative colitis-a pilot study to determine therapeutic benefit and graft stability. Gastroenterology. 2014;146(5):S-460.

247. De Fazio J, Kim S, Christley S, Fleming I, Shakhsheer B, Jabri B, et al. Replenishing the core gut microbiome via fecal microbiota transplant (FMT) can rescue mice from advanced-stage polymicrobial sepsis. Surgical Infections. 2014;15:S-10.

248. De Palma G, Lynch MD, Lu J, Deng Y, Jury J, Umeh G, et al. The adoptive transfer of anxiety and gut dysfunction from IBS patients to axenic mice through microbiota transplantation. Gastroenterology. 2014;146(5):S-845.

249. Didesch MM, Averill A, Oh-Park M. Peripheral neuropathy after fecal transplantation for clostridium difficile infection: A case report. PM and R. 2014;6(9):S237-S8.

250. Dodin M, Katz DE. Faecal microbiota transplantation for Clostridium difficile infection. Int J Clin Pract. 2014;68(3):363-8. pmid: 24372725 doi: 10.1111/ijcp.12320.

251. Dorottya NS, Faith I, Peera H, Abria H, Deborah S, Ann LR, et al. Mucosal disease activity may predict therapeutic response to fecal microbiome transplantation in pediatric ulcerative colitis. Inflamm Bowel Dis. 2014;20:S82-S3.

252. Drekonja DM. Clostridium difficile infection: Current, forgotten and emerging treatment options. J Comp Eff Res. 2014;3(5):547-57.

253. Dumitru IM, Dumitru E, Resul G, Curtali L, Paris S, Rugina S. Concomitant CMV and Clostridium difficile colitis in an immunocompetent patient treated with Ganciclovir and fecal transplantation. Journal of Gastrointestinal and Liver Diseases. 2014;23(2):221-2.

254. Ehlermann P, Dosch AO, Katus HA. Donor fecal transfer for recurrent Clostridium difficile-associated diarrhea in heart transplantation. J Heart Lung Transplant. 2014;33(5):551-3. pmid: 24742697 doi: 10.1016/j.healun.2014.02.002.

255. Emanuelsson F, Claesson BE, Ljungstrom L, Tvede M, Ung KA. Faecal microbiota transplantation and bacteriotherapy for recurrent Clostridium difficile infection: a retrospective evaluation of 31 patients. Scand J Infect Dis. 2014;46(2):89-97. pmid: 24354958 doi: 10.3109/00365548.2013.858181.

256. Freeman S, Mao E, Shah S, Kelly C. A case of recurrent clostridium difficile enteritis treated with fecal microbiota transplant. Am J Gastroenterol. 2014;109:S328.

257. Fuentes S, van Nood E, Tims S, Heikamp-de Jong I, ter Braak CJ, Keller JJ, et al. Reset of a critically disturbed microbial ecosystem: faecal transplant in recurrent Clostridium difficile infection. Isme j. 2014;8(8):1621-33. pmid: 24577353 doi: 10.1038/ismej.2014.13.

258. Ganc AJ, Ganc RL. Fecal microbiota transplantation, by means of push enteroscopy. A novel endoscopic technique, for the treatment of chronic diarrhea associated with clostridium difficile-a pilot study. Gastrointest Endosc. 2014;79(5):AB380-AB1.

259. Garg S, Song Y, Han MAT, Girotra M, Fricke WF, Dutta S. Post-infectious irritable bowel syndrome in patients undergoing fecal microbiota transplantation for recurrent clostridium difficile colitis. Gastroenterology. 2014;146(5):S83-S4.

260. Gordon H, Harbord M. A patient with severe Crohn's colitis responds to Faecal Microbiota Transplantation. J Crohns Colitis. 2014;8(3):256-7.

261. Greenwald D, Patel T, Barto A. Fecal microbiota transplant for treatment of refractory C. Difficile colitis: Long-term follow-up of 58 patients. Am J Gastroenterol. 2014;109:S679.

262. Greig J, Swope LK, Calvin H. Shaking up clostidium difficile infections: Implementation of a fecal microbiota transplant program. Am J Infect Control. 2014;42(6):S4-S5.

263. Guo WT, Wang JP, Liu P, Dong LN. New advances in clinical application of fecal microbiota transplantation. J Dig Dis. 2014;15:118-9.

264. Hargreaves KR, Clokie MRJ. Clostridium difficile phages: Still difficult? Front Microbiol. 2014;5(APR).

265. Hashash JG, Binion DG. Managing Clostridium difficile in Inflammatory Bowel Disease (IBD). Curr Gastroenterol Rep. 2014;16(7).

266. Hecht GA, Blaser MJ, Gordon J, Kaplan LM, Knight R, Laine L, et al. What is the value of a food and drug administration investigational new drug application for fecal microbiota transplantation to treat clostridium difficile infection? Clinical Gastroenterology and Hepatology. 2014;12(2):289-91.

267. Hintze KJ, Cox JE, Rompato G, Benninghoff AD, Ward RE, Broadbent J, et al. Broad scope method for creating humanized animal models for animal health and disease research through antibiotic treatment and human fecal transfer. . 2014;5(2):183-91. pmid: 24637796 doi: 10.4161/gmic.28403.

268. Högenauer C, Kump PK, Krause R. Tempered enthusiasm for fecal transplantation? Clinical Infectious Diseases. 2014;59(9):1348-9.

269. Hold GL, Smith M, Grange C, Watt ER, El-Omar EM, Mukhopadhya I. Role of the gut microbiota in inflammatory bowel disease pathogenesis: What have we learnt in the past 10 years? World J Gastroenterol. 2014;20(5):1192-210.

270. Honda H, Dubberke ER. The changing epidemiology of Clostridium difficile infection. Curr Opin Gastroenterol. 2014;30(1):54-62.

271. Ianiro G, Bibbo S, Scaldaferri F, Gasbarrini A, Cammarota G. Fecal microbiota transplantation in inflammatory bowel disease: beyond the excitement. Medicine (Baltimore). 2014;93(19):e97. pmid: 25340496 doi: 10.1097/md.0000000000000097.

272. Ianiro G, Gasbarrini A, Cammarota G. Letter: Faecal microbiota transplantation - Not a one-size-fits-all approach. Aliment Pharmacol Ther. 2014;40(1):119.

273. Jiang ZD, Ajami N, Lasco T, Petrosino J, Hochman F, Ankoma-Sey V, et al. Fresh, frozen, or lyophilized fecal microbiota transplantation (FMT) for multiple recurrent C. Difficile Infection (CDI). Am J Gastroenterol. 2014;109:S213.

274. Joseph J, Singhal S, Patel GM, Anand S. Clostridium difficile colitis: Review of the therapeutic approach. Am J Ther. 2014;21(5):385-94.

275. Kahn SA, Goeppinger SR, Rubin DT. Fecal microbiota transplantation: an interest in IBD? Nestle Nutr Inst Workshop Ser. 2014;79:101-14. pmid: 25227298 doi: 10.1159/000360715.

276. Kahn SA, Goeppinger SR, Vaughn BP, Moss AC, Rubin DT. Tolerability of colonoscopic fecal microbiota transplantation in IBD. Gastroenterology. 2014;146(5):S-581.

277. Kao D, Hotte N, Gillevet P, Madsen K. Fecal microbiota transplantation inducing remission in Crohn's colitis and the associated changes in fecal microbial profile. J Clin Gastroenterol. 2014;48(7):625-8. pmid: 24667590 doi: 10.1097/mcg.0000000000000131.

278. Kapel N, Thomas M, Corcos O, Mayeur C, Barbot-Trystram L, Bouhnik Y, et al. Practical implementation of faecal transplantation. Clin Microbiol Infect. 2014;20(11):1098-105. pmid: 25273614 doi: 10.1111/1469-0691.12796.

279. Karakan T. Fecal microbiota transplant in immunocompromised patients: Encouraging results in a vulnarable population. Turk J Gastroenterol. 2014;25(3):346.

280. Kassam Z, Lee CH, Hunt RH. Review of the emerging treatment of Clostridium difficile infection with fecal microbiota transplantation and insights into future challenges. Clin Lab Med. 2014;34(4):787-98. pmid: 25439277 doi: 10.1016/j.cll.2014.08.007.

281. Kazanowski M, Smolarek S, Kinnarney F, Grzebieniak Z. Clostridium difficile: Epidemiology, diagnostic and therapeutic possibilities - A systematic review. Tech Coloproctol. 2014;18(3):223-32.

282. Keller PM, Weber MH. Rational therapy of Clostridium difficile infections. Viszeralmedizin. 2014;30(5):304-9.

283. Kellermayer R, Nagy-Szakal D, Harris RA, Hollister EB, Mir S, Luna RA, et al. Clinical, epigenetic, and metagenomic responses to serial fecal microbiome transplants in pediatric ulcerative colitis. Gastroenterology. 2014;146(5):S-780.

284. Kelly CR, Kunde SS, Khoruts A. Guidance on preparing an investigational new drug application for fecal microbiota transplantation studies. Clin Gastroenterol Hepatol. 2014;12(2):283-8. pmid: 24107393 doi: 10.1016/j.cgh.2013.09.060.

285. Khan MA, Sofi AA, Ahmad U, Alaradi O, Khan AR, Hammad T, et al. Efficacy and safety of, and patient satisfaction with, colonoscopic-administered fecal microbiota transplantation in relapsing and refractory community- and hospital-acquired Clostridium difficile infection. Can J Gastroenterol Hepatol. 2014;28(8):434-8. pmid: 25014180.

286. Khanna S, Pardi DS. Clostridium difficile infection: Management strategies for a difficult disease. Therap Adv Gastroenterol. 2014;7(2):72-86.

287. Khoruts A. Faecal microbiota transplantation in 2013: developing human gut microbiota as a class of therapeutics. Nat Rev Gastroenterol Hepatol. 2014;11(2):79-80. pmid: 24296579 doi: 10.1038/nrgastro.2013.231.

288. Khoruts A, Weingarden AR. Emergence of fecal microbiota transplantation as an approach to repair disrupted microbial gut ecology. Immunology Letters. 2014.

289. Kim JE, Gweon TG, Yeo CD, Cho YS, Kim GJ, Kim JY, et al. A case of Clostridium difficile infection complicated by acute respiratory distress syndrome treated with fecal microbiota transplantation. World J Gastroenterol. 2014;20(35):12687-90. pmid: 25253977 doi: 10.3748/wjg.v20.i35.12687.

290. Koboziev I, Reinoso Webb C, Furr KL, Grisham MB. Role of the enteric microbiota in intestinal homeostasis and inflammation. Free Radic Biol Med. 2014;68:122-33.

291. Kump PK, Krause R, Allerberger F, Hogenauer C. Faecal microbiota transplantation--the Austrian approach. Clin Microbiol Infect. 2014;20(11):1106-11. pmid: 25274251 doi: 10.1111/1469-0691.12801.

292. Lagier JC. Faecal microbiota transplantation: from practice to legislation before considering industrialization. Clin Microbiol Infect. 2014;20(11):1112-8. pmid: 25273480 doi: 10.1111/1469-0691.12795.

293. Le Monnier A, Zahar JR, Barbut F. Update on Clostridium difficile infections. Medecine et Maladies Infectieuses. 2014.

294. Ley RE. Harnessing microbiota to kill a pathogen: the sweet tooth of Clostridium difficile. Nat Med. 2014;20(3):248-9. pmid: 24603797 doi: 10.1038/nm.3494.

295. Li Q, Wang C, Tang C, He Q, Zhao X, Li N, et al. Therapeutic modulation and reestablishment of the intestinal Microbiota with fecal Microbiota transplantation resolves sepsis and diarrhea in a patient. Am J Gastroenterol. 2014;109(11):1832-4.

296. Liang J, Sha SM, Wu KC. Role of the intestinal microbiota and fecal transplantation in inflammatory bowel diseases. J Dig Dis. 2014;15(12):641-6. pmid: 25389085 doi: 10.1111/1751-2980.12211.

297. Libertucci J, Whelan FJ, Moayyedi P, Lee CH, Wolfe M, Onishi C, et al. Investigating the microbiome pre and post fecal microbiota therapy from active ulcerative colitis patients in a randomized placebo controlled trial. Gastroenterology. 2014;146(5):S-902.

298. Lingala S. Fecal microbiota transplantation in critically ill patient with severe clostridium difficile colitis. Gastroenterology. 2014;146(5):S-251.

299. Lo Vecchio A, Cohen MB. Fecal microbiota transplantation for Clostridium difficile infection: benefits and barriers. Curr Opin Gastroenterol. 2014;30(1):47-53. pmid: 24275671 doi: 10.1097/mog.0000000000000023.

300. Lofgren ET, Moehring RW, Anderson DJ, Weber DJ, Fefferman NH. A mathematical model to evaluate the routine use of fecal microbiota transplantation to prevent incident and recurrent Clostridium difficile infection. Infect Control Hosp Epidemiol. 2014;35(1):18-27. pmid: 24334794 doi: 10.1086/674394.

301. Long Miao C, Mowery A, Khara H, Shellenberger M, Komar M. C. difficile enteritis after total proctocolectomy successfully treated with fecal transplant. Am J Gastroenterol. 2014;109:S442.

302. Mantzaris GJ. When can we cure Crohn's? Best Pract Res Clin Gastroenterol. 2014;28(3):519-29.

303. Massachi S, Hay JW. Cost-effectiveness of various clostridium difficile infection (CDI) treatments in patients with recurrent infections. Value in Health. 2014;17(3):A273-A4.

304. Matsuoka K, Mizuno S, Hayashi A, Hisamatsu T, Naganuma M, Kanai T. Fecal microbiota transplantation for gastrointestinal diseases. Keio J Med. 2014;63(4):69-74. pmid: 25500625 doi: 10.2302/kjm.2014-0006-RE.

305. McCune VL, Struthers JK, Hawkey PM. Faecal transplantation for the treatment of Clostridium difficile infection: A review. Int J Antimicrob Agents. 2014;43(3):201-6.

306. Megerlin F, Fouassier E, Lopert R, Bourlioux P. Faecal microbiota transplantation: a sui generis biological drug, not a tissue. Ann Pharm Fr. 2014;72(4):217-20. pmid: 24997882 doi: 10.1016/j.pharma.2014.04.008.

307. Merenstein D, El-Nachef N, Lynch SV. Fecal microbial therapy: promises and pitfalls. J Pediatr Gastroenterol Nutr. 2014;59(2):157-61. pmid: 24796803 doi: 10.1097/mpg.0000000000000415.

308. Mirshahi S, Puri P, Pacana T, Daita K, Patel V, Min HK, et al. Vitamin e alters intestinal microbiome through differential changes in mucosal and fecal microbiota in mouse model of nonalcoholic fatty liver disease (NALFD). J Hepatol. 2014;60(1):S164.

309. Moayyedi P, Marshall JK, Yuan Y, Hunt R. Canadian Association of Gastroenterology position statement: fecal microbiota transplant therapy. Can J Gastroenterol Hepatol. 2014;28(2):66-8. pmid: 25232572.

310. Moayyedi P, Surette M, Wolfe M, Taraschi R, Kim P, Libertucci J, et al. A randomized, placebo controlled trial of fecal microbiota therapy in active ulcerative colitis. Gastroenterology. 2014;146(5):S-159.

311. Montassier E, Batard E, Massart S, Gastinne T, Carton T, Caillon J, et al. 16S rRNA gene pyrosequencing reveals shift in patient faecal microbiota during high-dose chemotherapy as conditioning regimen for bone marrow transplantation. Microb Ecol. 2014;67(3):690-9. pmid: 24402367 doi: 10.1007/s00248-013-0355-4.

312. Moore T, Rodriguez A, Bakken JS. Fecal microbiota transplantation: a practical update for the infectious disease specialist. Clin Infect Dis. 2014;58(4):541-5. pmid: 24368622 doi: 10.1093/cid/cit950.

313. Muniesa M, Jofre J. Identifying and analyzing bacteriophages in human fecal samples: what could we discover? Future Microbiol. 2014;9(7):879-86. pmid: 25156377 doi: 10.2217/fmb.14.47.

314. Nieuwdorp M. Faecal microbiota transplantation. Br J Surg. 2014;101(8):887-8. pmid: 24844698 doi: 10.1002/bjs.9549.

315. O'Horo JC, Jindai K, Kunzer B, Safdar N. Treatment of recurrent Clostridium difficile infection: A systematic review. Infection. 2014;42(1):43-59.

316. Pamer EG. Fecal microbiota transplantation: effectiveness, complexities, and lingering concerns. Mucosal Immunol. 2014;7(2):210-4. pmid: 24399149 doi: 10.1038/mi.2013.117.

317. Paramsothy S, Borody T, Lin E, Finlayson S, Walsh A, Samuel D, et al. Obstacles to donor recruitment for faecal microbiota transplantation-Experiences from the FOCUS study. J Gastroenterol Hepatol (Australia). 2014;29:135.

318. Park L, Tzimas D, Price J, Mone A, Hirsh J, Poles M, et al. Perceptions of fecal microbiota transplantation: Factors that predict acceptance: A preliminary analysis. Am J Gastroenterol. 2014;109:S206.

319. Patel LN, Schairer J, Shen B. Fecal transplantation therapy for Clostridium difficile-associated pouchitis. Int J Colorectal Dis. 2014;29(2):263-4.

320. Peer X, An G. Agent-based model of fecal microbial transplant effect on bile acid metabolism on suppressing Clostridium difficile infection: an example of agent-based modeling of intestinal bacterial infection. J Pharmacokinet Pharmacodyn. 2014;41(5):493-507. pmid: 25168489 PubMed Central PMCID: PMCPmc4210368 doi: 10.1007/s10928-014-9381-1.

321. Petrof EO, Khoruts A. From stool transplants to next-generation microbiota therapeutics. Gastroenterology. 2014;146(6):1573-82. pmid: 24412527 doi: 10.1053/j.gastro.2014.01.004.

322. Pires RN, Monteiro AA, Carneiro LC, Baethgen LF, Tavares R, Lincho CS, et al. Clostridium difficile infection in Brazil: A neglected problem? Am J Infect Control. 2014;42(4):459-60.

323. Polák P, Husa P, Freibergerová M. Colitis due to Clostridium difficile in broader context. Interni Medicina pro Praxi. 2014;16(6):241-4.

324. Rabe SM. Treatment of recurrent Clostridium difficile infection with fecal transplantation. Gastroenterol Nurs. 2014;37(2):156-63; quiz 64-5. pmid: 24691086 doi: 10.1097/sga.0000000000000035.

325. Raghunath V, Levy M, Koo K, Foo H, Borody TJ, Wong J. Recurrent clostridium difficile infection in a renal transplant recipient successfully treated with fecal microbiota transplantation. Nephrology. 2014;19:98.

326. Rao K, Young VB, Aronoff DM. Fecal microbiota therapy: Ready for prime time? Infect Control Hosp Epidemiol. 2014;35(1):28-30.

327. Raoult D. Faecal transplantation and infectious diseases practitioners. Clin Microbiol Infect. 2014;20(11):1097.

328. Ratner M. Fecal transplantation poses dilemma for FDA. Nature biotechnology. 2014;32(5):401-2.

329. Ren RR, Sun G, Ban YL, Yang YS. The Chinese physicians' perception of fecal microbiota transplantation. J Dig Dis. 2014;15:152-3.

330. Ren RR, Sun G, Peng LH, Wang ZK, Yang YS. Pilot study of treating refractory inflammation bowel disease with fecal microbiota transplantation. J Dig Dis. 2014;15:153.

331. Rineh A, Kelso MJ, Vatansever F, Tegos GP, Hamblin MR. Clostridium difficile infection: Molecular pathogenesis and novel therapeutics. Expert Rev Anti Infect Ther. 2014;12(1):131-50.

332. Roehr B. FDA faces regulatory challenges with new approaches to medicine. BMJ (Online). 2014;348.

333. Rupali P, Mittal C, Deol A, Alangaden G, Ramesh M. Fecal microbiota transplantation for clostridium difficile infection in immunocompromised hosts: One easy strategy, one giant success. Transplantation. 2014;98:687-8.

334. Sadowsky MJ, Weingarden A, Khoruts A, Gonzalez A, Vázquez-Baeza Y, Weiss S, et al. Short and long term changes in bacterial composition following fecal microbiota transplantation for CDI visualized in movie format. Gastroenterology. 2014;146(5):S-838.

335. Sageer M, Barto A. Recurrent Clostridium difficile infection: The scope of the problem and management decisions. Semin Colon Rectal Surg. 2014;25(3):158-62.

336. Sammons JS, Gerber JS, Tamma PD, Sandora TJ, Beekmann SE, Polgreen PM, et al. Diagnosis and management of Clostridium difficile infection by pediatric infectious diseases physicians. J Pediatric Infect Dis Soc. 2014;3(1):43-8.

337. Samuel BP, Crumb TL, Duba MM. What nurses need to know about fecal microbiota transplantation: education, assessment, and care for children and young adults. J Pediatr Nurs. 2014;29(4):354-61. pmid: 24582649 doi: 10.1016/j.pedn.2014.01.013.

338. Schenck LP, Hirota S, Armstrong G, MacDonald J, Beck P. Investigating the effect of antibiotics on gut microbiota components and subsequent Clostridium difficile infection. FASEB Journal. 2014;28(1).

339. Schünemann M, Oette M. Fecal microbiota transplantation for Clostridium difficile-associated colitis in a severely immunocompromized critically ill AIDS patient: A case report. AIDS. 2014;28(5):798-9.

340. Seekatz AM, Aas J, Gessert CE, Rubin TA, Saman DM, Bakken JS, et al. Recovery of the gut microbiome following fecal microbiota transplantation. MBio. 2014;5(3):e00893-14. pmid: 24939885 doi: 10.1128/mBio.00893-14.

341. Seril DN, Shen B. Clostridium difficile infection in the postcolectomy patient. Inflamm Bowel Dis. 2014;20(12):2450-69.

342. Sha S, Liang J, Chen M, Xu B, Liang C, Wei N, et al. Systematic review: faecal microbiota transplantation therapy for digestive and nondigestive disorders in adults and children. Aliment Pharmacol Ther. 2014;39(10):1003-32. pmid: 24641570 doi: 10.1111/apt.12699.

343. Sha S, Wu K. Letter: Faecal microbiota transplantation - Not a one-size-fits-all approach; Authors' reply. Aliment Pharmacol Ther. 2014;40(1):119-20.

344. Shanahan F, Quigley EMM. Manipulation of the microbiota for treatment of IBS and IBD - Challenges and controversies. Gastroenterology. 2014;146(6):1554-63.

345. Singh R, Nieuwdorp M, ten Berge IJ, Bemelman FJ, Geerlings SE. The potential beneficial role of faecal microbiota transplantation in diseases other than Clostridium difficile infection. Clin Microbiol Infect. 2014;20(11):1119-25. pmid: 25274035 doi: 10.1111/1469-0691.12799.

346. Singh R, van Nood E, Nieuwdorp M, van Dam B, ten Berge IJ, Geerlings SE, et al. Donor feces infusion for eradication of Extended Spectrum beta-Lactamase producing Escherichia coli in a patient with end stage renal disease. Clin Microbiol Infect. 2014;20(11):O977-8. pmid: 24845223 doi: 10.1111/1469-0691.12683.

347. Smith MB, Kelly C, Alm EJ. How to regulate faecal transplants. Nature. 2014;506(7488):290-1.

348. Solari PR, Fairchild PG, Noa LJ, Wallace MR. Tempered enthusiasm for fecal transplant. Clinical Infectious Diseases. 2014;59(2):319.

349. Stysly B, Kukkadapu T, Singh E. Clostridium difficile in ulcerative colitis complicated by underlying aplastic anemia. Am J Gastroenterol. 2014;109:S443.

350. Suskind D, Wahbeh G, Vendetoulli H, Singh N, Miller S. Fecal microbial transplant in pediatric crohn's disease. Gastroenterology. 2014;146(5):S-834.

351. Swaminath A. The power of poop: Patients getting ahead of their doctors using self-administered fecal transplants. Am J Gastroenterol. 2014;109(5):777-8.

352. Taur Y, Pamer EG. Harnessing microbiota to kill a pathogen: Fixing the microbiota to treat Clostridium difficile infections. Nature Medicine. 2014;20(3):246-7.

353. Tian P, Xu B, Sun H, Li X, Li Z, Wei P. Isolation and gut microbiota modulation of antibiotic-resistant probiotics from human feces. Diagn Microbiol Infect Dis. 2014;79(4):405-12. pmid: 24820193 doi: 10.1016/j.diagmicrobio.2014.04.002.

354. To KB, Napolitano LM. Clostridium difficile infection: Update on diagnosis, epidemiology, and treatment strategies. Surgical Infections. 2014;15(5):490-502.

355. True E, Tsoraides S, Wang H, Farrell J, Bonello J. Predictors of failure with fecal microbiota therapy for recurrent clostridium difficile colitis. Dis Colon Rectum. 2014;57(5):e99-e100.

356. Tsutsumi LS, Owusu YB, Hurdle JG, Sun D. Progress in the discovery of treatments for C. difficile infection: A clinical and medicinal chemistry review. Curr Top Med Chem. 2014;14(1):152-75.

357. Udayappan SD, Hartstra AV, Dallinga-Thie GM, Nieuwdorp M. Intestinal microbiota and faecal transplantation as treatment modality for insulin resistance and type 2 diabetes mellitus. Clin Exp Immunol. 2014;177(1):24-9. pmid: 24528224 doi: 10.1111/cei.12293.

358. Vaishnavi C. Fecal microbiota transplantation for management of Clostridium difficile infection. Indian J Gastroenterol. 2014;33(4):301-7. pmid: 24748025 doi: 10.1007/s12664-014-0459-x.

359. Valeur J, Midtvedt T. Faeces transplantation-new wonder medicine? Tidsskr Nor Laegeforen. 2014;134(23-24):2233. pmid: 25492317 doi: 10.4045/tidsskr.14.1261.

360. van Nood E, Speelman P, Nieuwdorp M, Keller J. Fecal microbiota transplantation: facts and controversies. Curr Opin Gastroenterol. 2014;30(1):34-9. pmid: 24241245 doi: 10.1097/mog.0000000000000024.

361. Varier RU, Biltaji EO, Smith KJ, Roberts MS, LaFleur J, Nelson RE. Cost-effectiveness analysis of fecal microbiota transplantation versus vancomycin for recurrent clostridium difficile infection. Gastroenterology. 2014;146(5):S250-S1.

362. Vaughn BP, Gevers D, Ting A, Korzenik JR, Robson SC, Moss AC. Fecal microbiota transplantation induces early improvement in symptoms in patients with active crohn's disease. Gastroenterology. 2014;146(5):S591-S2.

363. Veling N. A novel approach in the treatment of clostridium difficile: A case study. J Spinal Cord Med. 2014;37(4):441-2.

364. Verbeke KA, Boesmans L, Boets E. Modulating the microbiota in inflammatory bowel diseases: Prebiotics, probiotics or faecal transplantation? Proc Nutr Soc. 2014;73(4):490-7.

365. Walia R, Garg S, Song Y, Girotra M, Cuffari C, Fricke WF, et al. Efficacy of fecal microbiota transplantation in 2 children with recurrent Clostridium difficile infection and its impact on their growth and gut microbiome. J Pediatr Gastroenterol Nutr. 2014;59(5):565-70. pmid: 25023578 doi: 10.1097/mpg.0000000000000495.

366. Walia R, Kunde S, Mahajan L. Fecal microbiota transplantation in the treatment of refractory Clostridium difficile infection in children: an update. Curr Opin Pediatr. 2014;26(5):573-8. pmid: 25046331 doi: 10.1097/mop.0000000000000127.

367. Wang ZK, Yang YS, Chen Y, Yuan J, Sun G, Peng LH. Intestinal microbiota pathogenesis and fecal microbiota transplantation for inflammatory bowel disease. World J Gastroenterol. 2014;20(40):14805-20. pmid: 25356041 doi: 10.3748/wjg.v20.i40.14805.

368. Ward MA, Alverdy JC, Chang EB. Microbes modified by luminal stasis play a central role in the development of ulcerative colitis. J Surg Res. 2014;186(2):625.

369. Weingarden AR, Chen C, Bobr A, Yao D, Lu Y, Nelson VM, et al. Microbiota transplantation restores normal fecal bile acid composition in recurrent clostridium difficile infection. Clinical and Translational Science. 2014;7(3):231.

370. Wittebole X, De Roock S, Opal SM. A historical overview of bacteriophage therapy as an alternative to antibiotics for the treatment of bacterial pathogens. Virulence. 2014;5(1):209-18.

371. Yang J, Rose DJ. Long-term dietary pattern of fecal donor correlates with butyrate production and markers of protein fermentation during in vitro fecal fermentation. Nutr Res. 2014;34(9):749-59. pmid: 25218569 doi: 10.1016/j.nutres.2014.08.006.

372. Youngster I, Sauk J, Pindar C, Wilson RG, Kaplan JL, Smith MB, et al. Fecal microbiota transplant for relapsing Clostridium difficile infection using a frozen inoculum from unrelated donors: a randomized, open-label, controlled pilot study. Clin Infect Dis. 2014;58(11):1515-22. pmid: 24762631 doi: 10.1093/cid/ciu135.

373. Zalig S, Rupnik M. Clostridium difficile infection and gut microbiota. Semin Colon Rectal Surg. 2014;25(3):124-7.

374. Zanella Terrier MC, Simonet ML, Bichard P, Frossard JL. Recurrent Clostridium difficile infections: The importance of the intestinal microbiota. World J Gastroenterol. 2014;20(23):7416-23.

375. Zeller G, Tap J, Voigt AY, Sunagawa S, Kultima JR, Costea PI, et al. Potential of fecal microbiota for early-stage detection of colorectal cancer. Mol Syst Biol. 2014;10:766. pmid: 25432777 doi: 10.15252/msb.20145645.

376. Zipursky JS, Sidorsky TI, Freedman CA, Sidorsky MN, Kirkland KB. Physician attitudes toward the use of fecal microbiota transplantation for the treatment of recurrent Clostridium difficile infection. Can J Gastroenterol Hepatol. 2014;28(6):319-24. pmid: 24719899.

377. Agilli M, Ilga U. A methodological approach to fecal microbial transplant via nasogastric tube for active pediatric ulcerative colitis. J Pediatr Gastroenterol Nutr. 2015;60(4):e36. pmid: 25822774 doi: 10.1097/mpg.0000000000000716.

378. Allegretti JR, Hamilton MJ, Korzenik JR, Chan WW. Factors associated with C. Difficile negative gastrointestinal symptoms after intestinal microbiome restoration. Gastroenterology. 2015;148(4):S643.

379. Anand R, Song Y, Sinha A, Hasan S, Sivaraman A, Garg S, et al. Effect of aging on the fecal microbiome in healthy donors for fecal microbiota transplant. Gastroenterology. 2015;148(4):S719.

380. Aratari A, Cammarota G, Papi C. Fecal microbiota transplantation for recurrent C. difficile infection in a patient with chronic refractory ulcerative colitis. J Crohns Colitis. 2015;9(4):367. pmid: 25687205 doi: 10.1093/ecco-jcc/jjv034.

381. Armstrong MJ, Pathmakanthan S, Iqbal TH. Fecal microbiota transplantation for Clostridium difficile infection. JAMA. 2015;313(7):725-6.

382. Bagdasarian N, Rao K, Malani PN. Diagnosis and treatment of clostridium difficile in adults: A systematic review. JAMA. 2015;313(4):398-408.

383. Baxter M, Ahmad T, Colville A, Sheridan R. Fatal Aspiration Pneumonia as a Complication of Fecal Microbiota Transplant. Clin Infect Dis. 2015;61(1):136-7. pmid: 25805303 doi: 10.1093/cid/civ247.

384. Borgia G, Maraolo AE, Foggia M, Buonomo AR, Gentile I. Fecal microbiota transplantation for Clostridium difficile infection: back to the future. Expert Opin Biol Ther. 2015;15(7):1001-14. pmid: 26063385 doi: 10.1517/14712598.2015.1045872.

385. Bourlioux P. Faecal microbiota transplantation: Key points to consider. Ann Pharm Fr. 2015;73(3):163-8.

386. Brechmann T, Swol J, Knop-Hammad V, Willert J, Aach M, Cruciger O, et al. Complicated fecal microbiota transplantation in a tetraplegic patient with severe Clostridium difficile infection. World J Gastroenterol. 2015;21(12):3736-40. pmid: 25834343 doi: 10.3748/wjg.v21.i12.3736.

387. Buffie CG, Bucci V, Stein RR, McKenney PT, Ling L, Gobourne A, et al. Precision microbiome reconstitution restores bile acid mediated resistance to Clostridium difficile. Nature. 2015;517(7533):205-8. pmid: 25337874 doi: 10.1038/nature13828.

388. Burns LJ, Dubois N, Smith MB, Mendolia GM, Burgess J, Edelstein C, et al. Donor recruitment and eligibility for fecal microbiota transplantation: Results from an international public stool bank. Gastroenterology. 2015;148(4):S96-S7.

389. Cohen NA, Ben Ami R, Guzner-Gur H, Santo ME, Halpern Z, Maharshak N. Fecal Microbiota Transplantation for Clostridium difficile-Associated Diarrhea. Isr Med Assoc J. 2015;17(8):510-4. pmid: 26394495.

390. Costello ME, Robinson PC, Benham H, Brown MA. The intestinal microbiome in human disease and how it relates to arthritis and spondyloarthritis. Best Pract Res Clin Rheumatol. 2015;29(2):202-12. pmid: 26362739 doi: 10.1016/j.berh.2015.08.001.

391. Costello SP, Chung A, Andrews JM, Fraser RJ. Fecal microbiota transplant for clostridium difficile colitis-induced toxic megacolon. Am J Gastroenterol. 2015;110(5):775-7.

392. Crum-Cianflone NF, Sullivan E, Ballon-Landa G. Fecal microbiota transplantation and successful resolution of multidrug-resistant-organism colonization. J Clin Microbiol. 2015;53(6):1986-9. pmid: 25878340 doi: 10.1128/jcm.00820-15.

393. Dai C, Jiang M, Sun MJ. Can fecal microbial transplant effectively treat Crohn's disease? Inflamm Bowel Dis. 2015;21(6):E8.

394. Dai C, Jiang M, Sun MJ. Fecal microbiota transplantation for treatment of clostridium difficile infection. J Clin Gastroenterol. 2015;49(2):171-2.

395. Dakhoul L, Parikh K, Berkelhammer C. Fecal microbiota transplant in treatment of clostridium difficile colitis-pooled data analysis and a systematic review. Gastroenterology. 2015;148(4):S404.

396. Damman CJ, Brittnacher MJ, Westerhoff M, Hayden HS, Radey M, Hager KR, et al. Low Level Engraftment and Improvement following a Single Colonoscopic Administration of Fecal Microbiota to Patients with Ulcerative Colitis. PLoS One. 2015;10(8):e0133925. pmid: 26288277 doi: 10.1371/journal.pone.0133925.

397. De Castro CG, Ganc AJ, Ganc RL, Petrolli MS, Hamerschlack N. Fecal microbiota transplant after hematopoietic SCT: Report of a successful case. Bone Marrow Transplantation. 2015;50(1):145.

398. Dennis M, Salpeter MJ, Hota S. Low awareness but positive attitudes toward fecal transplantation in ontario physicians. Can J Infect Dis Med Microbiol. 2015;26(1):30-2.

399. Di Bella S, Gouliouris T, Petrosillo N. Fecal microbiota transplantation (FMT) for Clostridium difficile infection: focus on immunocompromised patients. J Infect Chemother. 2015;21(4):230-7. pmid: 25703532 doi: 10.1016/j.jiac.2015.01.011.

400. Drekonja D, Reich J, Gezahegn S, Greer N, Shaukat A, MacDonald R, et al. Fecal Microbiota Transplantation for Clostridium difficile Infection: A Systematic Review. Ann Intern Med. 2015;162(9):630-8. pmid: 25938992 doi: 10.7326/m14-2693.

401. Duke PS, Fardy J. Recurrent Clostridium difficile infection treated with home fecal transplantation: A case report. J Med Case Rep. 2015:1-4.

402. Fischer M, Sipe BW, Rogers NA, Cook GK, Robb BW, Vuppalanchi R, et al. Faecal microbiota transplantation plus selected use of vancomycin for severe-complicated Clostridium difficile infection: Description of a protocol with high success rate. Aliment Pharmacol Ther. 2015.

403. Ganc AJ, Ganc RL, Reimao SM, Frisoli Junior A, Pasternak J. Fecal microbiota transplant by push enteroscopy to treat diarrhea caused by Clostridium difficile. Einstein (Sao Paulo). 2015;13(2):338-9. pmid: 26154556 doi: 10.1590/s1679-45082015md3106.

404. Gillard L, Mayeur C, Lepage P, Miquel S, Robert V, Beyec- Le Bihan JL, et al. Short bowel syndrome: A case study to illustrate how gut remodeling can impact fecal microbiota that in turn becomes central to understanding clinical outcome. Gastroenterology. 2015;148(4):S82-S3.

405. Gregory JC, Buffa JA, Org E, Wang Z, Levison BS, Zhu W, et al. Transmission of atherosclerosis susceptibility with gut microbial transplantation. J Biol Chem. 2015;290(9):5647-60. pmid: 25550161 doi: 10.1074/jbc.M114.618249.

406. Grinspan AM, Kelly CR. Fecal Microbiota Transplantation for Ulcerative Colitis: Not Just Yet. Gastroenterology. 2015;149(1):15-8.

407. Gundling F, Tiller M, Agha A, Schepp W, Iesalnieks I. Successful autologous fecal transplantation for chronic diversion colitis. Tech Coloproctol. 2015;19(1):51-2. pmid: 25300242 doi: 10.1007/s10151-014-1220-2.

408. Gweon TG, Lee KJ, Kang DH, Park SS, Kim KH, Seong HJ, et al. A case of toxic megacolon caused by clostridium difficile infection and treated with fecal microbiota transplantation. Gut Liver. 2015;9(2):247-50. pmid: 25721003 PubMed Central PMCID: PMCPmc4351033 doi: 10.5009/gnl14152.

409. Hirsch BE, Saraiya N, Poeth K, Schwartz RM, Epstein ME, Honig G. Effectiveness of fecal-derived microbiota transfer using orally administered capsules for recurrent Clostridium difficile infection. BMC Infect Dis. 2015;15:191. pmid: 25885020 doi: 10.1186/s12879-015-0930-z.

410. Ho JT, Chan GC, Li JC. Systemic effects of gut microbiota and its relationship with disease and modulation. BMC Immunol. 2015;16:21. pmid: 25896342 doi: 10.1186/s12865-015-0083-2.

411. Holvoet T, Boelens J, Joossens M, Raes J, De Vos M, De Looze D. Fecal microbiota transplantation in irritable bowel syndrome with bloating: Results from a prospective pilot study. Gastroenterology. 2015;148(4):S963-S4.

412. Hourigan S, Ann Chen L, Grigoryan Z, Laroche G, Weidner M, Sears CL, et al. Microbiome changes associated with sustained eradication of clostridium difficile after fecal microbiota transplantation in children with and without inflammatory bowel disease. Gastroenterology. 2015;148(4):S45.

413. Hubble L, Joshua S, Glover PH, Trivedi A, Pfanner TP. Colonoscopic vs. Upper endoscopic placement of fecal microbiota transplant for recurrent clostridium difficile infection: A retrospective review. Gastroenterology. 2015;148(4):S728.

414. Jain A, Parian AM, Dudley-Brown S, Lazarev M. Fecal microbiota transplantation is safe and effective for treatment of recurrent clostridium difficile infection in inflammatory bowel disease patients. Gastroenterology. 2015;148(4):S869.

415. Jones JD, Murphy DW. Rescue fecal microbiota transplantation in refractory severe and complicated clostridium difficile infection using frozen stool specimens. Gastroenterology. 2015;148(4):S641.

416. Jones L, Jones C. Does the donor matter? Donor vs. patient effects in the outcome of next-generation fecal transplant for recurrent clostridium difficile infection. Gastroenterology. 2015;148(4):S328-S9.

417. Kellermayer R, Hollister EB, Nagy-Szakal D, Ihekweazu FD, Haynes A, Pitashny M, et al. Special considerations for fecal microbiota transplantation in pediatric recurrent clostridium difficile infection. Gastroenterology. 2015;148(4):S961-S2.

418. Kellermayer R, Nagy-Szakal D, Harris RA, Luna RA, Pitashny M, Schady D, et al. Serial fecal microbiota transplantation alters mucosal gene expression in pediatric ulcerative colitis. Am J Gastroenterol. 2015;110(4):604-6.

419. Kelly CR. Editorial: A simple faecal preparation protocol for faecal microbiota transplantation. Aliment Pharmacol Ther. 2015;41(3):320.

420. Kelly CR, Kahn S, Kashyap P, Laine L, Rubin D, Atreja A, et al. Update on Fecal Microbiota Transplantation 2015: Indications, Methodologies, Mechanisms, and Outlook. Gastroenterology. 2015;149(1):223-37.

421. Khanna S, Weatherly RM, Kammer PP, Loftus EV, Pardi DS. Long-term follow-up after fecal microbiota transplantation for C. Difficile infection in inflammatory bowel disease patients. Gastroenterology. 2015;148(4):S726.

422. Khoruts A, Rank KM, Viskocil K, Newman KM. Diagnostic value of colonoscopy in patients receiving fecal microbiota transplantation in treatment of refractory clostridium difficile infection. Gastroenterology. 2015;148(4):S729.

423. Khoruts A, Sadowsky MJ, Hamilton MJ. Development of fecal microbiota transplantation suitable for mainstream medicine. Clin Gastroenterol Hepatol. 2015;13(2):246-50. pmid: 25460566 doi: 10.1016/j.cgh.2014.11.014.

424. Kim YG, Jang BI. Current advances related to Clostridium difficile infection. Indian J Med Res. 2015;142(FEB):172-4.

425. Konturek PC, Haziri D, Brzozowski T, Hess T, Heyman S, Kwiecien S, et al. Emerging role of fecal microbiota therapy in the treatment of gastrointestinal and extra-gastrointestinal diseases. J Physiol Pharmacol. 2015;66(4):483-91. pmid: 26348073.

426. Korman TM. Diagnosis and Management of Clostridium difficile Infection. Semin Respir Crit Care Med. 2015;36(1):31-43.

427. Kuperman AA, Koren O. The Era of Fecal Microbiota Transplantation. Isr Med Assoc J. 2015;17(8):515-6. pmid: 26394496.

428. Lagier JC, Delord M, Million M, Parola P, Stein A, Brouqui P, et al. Dramatic reduction in Clostridium difficile ribotype 027-associated mortality with early fecal transplantation by the nasogastric route: a preliminary report. Eur J Clin Microbiol Infect Dis. 2015;34(8):1597-601. pmid: 25947205 doi: 10.1007/s10096-015-2394-x.

429. Lagier JC, Million M, Fournier PE, Brouqui P, Raoult D. Faecal microbiota transplantation for stool decolonization of OXA-48 carbapenemase-producing Klebsiella pneumoniae. J Hosp Infect. 2015;90(2):173-4.

430. Lee WJ, Lattimer LDN, Stephen S, Borum ML, Doman DB. Fecal microbiota transplantation: A review of emerging indications beyond relapsing Clostridium difficile toxin colitis. Gastroenterology and Hepatology. 2015;11(1):24-32.

431. Leis S, Borody TJ, Jiang C, Campbell J. Fecal microbiota transplantation: A 'How-To' guide for nurses. Collegian. 2015;22(4):445-51. pmid: 26775532.

432. Li Q, Wang C, Tang C, He Q, Zhao X, Li N, et al. Successful treatment of severe sepsis and diarrhea after vagotomy utilizing fecal microbiota transplantation: a case report. Crit Care. 2015;19:37. pmid: 25881250 doi: 10.1186/s13054-015-0738-7.

433. Lin E, Jaworski A, Furnari V, Wong C, Bull M, Chapman B, et al. Twelve week storage trial of microbial viability in lyophilized and frozen fecal microbiota preparations. Gastroenterology. 2015;148(4):S962.

434. Lynch SV. Fecal microbiota transplantation for recurrent clostridium difficile infection in pediatric patients: Encouragement wrapped in caution. J Pediatr Gastroenterol Nutr. 2015;60(1):1-3.

435. Mansfield LS, Brakel KA, Malik A, Bell JA. A human microbiome enhanced campylobacter jejuni induced autoantibodies and Th-2 skewing of adaptive immunity after fecal transplant. J Allergy Clin Immunol. 2015;135(2):AB99.

436. Marcos LA, Gersh A, Blanchard K, Foil S, Mallini B, Farrell SE, et al. Fecal transplantation to treat initial severe Clostridium difficile infection with sepsis. J Miss State Med Assoc. 2015;56(2):38-40. pmid: 25895282.

437. Matuchansky C. Fecal microbiota transplantation: The case of immunocompromised patients. Am J Med. 2015;128(3):e21.

438. Millan B, Hotte N, Mathieu O, Burguiere P, Tompkins TA, Kao D, et al. Effects of fecal microbial transplantation on the gut resistome in patients with recurrent clostridium difficile infection. Gastroenterology. 2015;148(4):S120.

439. Million M, Hocquart M, Seghboyan JM, Griffiths K, Halfon P, Lagier JC, et al. Faecal microbiota transplantation as salvage therapy for fulminant Clostridium difficile infections. Int J Antimicrob Agents. 2015;46(2):227-8. pmid: 26092095 doi: 10.1016/j.ijantimicag.2015.05.002.

440. Mittal C, John A, Hart BR, Miller N, Meighani A, Ramesh M. Fecal microbiota transplantation for recurrent and/or refractory clostridium difficile infection: A large retrospective study of failure rates, predictors of failure and outcomes. Gastroenterology. 2015;148(4):S723-S4.

441. Mittal C, Miller N, Meighani A, Hart BR, John A, Ramesh M. Fecal microbiota transplant for recurrent Clostridium difficile infection after peripheral autologous stem cell transplant for diffuse large B-cell lymphoma. Bone Marrow Transplantation. 2015;50(7):1010.

442. Mullish BH, Marchesi JR, Thursz MR, Williams HR. Microbiome manipulation with faecal microbiome transplantation as a therapeutic strategy in Clostridium difficile infection. Qjm. 2015;108(5):355-9. pmid: 25193538 doi: 10.1093/qjmed/hcu182.

443. Nieuwdorp M. Fecal transplantation in obesity and metabolic syndrome. Obesity Facts. 2015;8:6.

444. Olefson SH, Jackson M, Kelly C. Clostridium difficile: The spectrum of diagnoses in patients referred for fecal microbiota transplant. Gastroenterology. 2015;148(4):S727.

445. Orduña P, Lopez SY, Schmulson M, Arredondo R, de Leon SP, Lopez-Vidal Y. A survey using the social networks revealed poor knowledge on fecal microbiota transplantation. J Neurogastroenterol Motil. 2015;21(2):294-5.

446. Paramsothy S, Borody TJ, Lin E, Finlayson S, Walsh AJ, Samuel D, et al. Donor Recruitment for Fecal Microbiota Transplantation. Inflamm Bowel Dis. 2015;21(7):1600-6. pmid: 26070003 doi: 10.1097/mib.0000000000000405.

447. Pinn DM, Aroniadis OC, Brandt LJ. Is fecal microbiota transplantation (FMT) an effective treatment for patients with functional gastrointestinal disorders (FGID)? Neurogastroenterol Motil. 2015;27(1):19-29. pmid: 25424663 doi: 10.1111/nmo.12479.

448. Porter RJ. Pulsed faecal microbiota transplantation for recalcitrant recurrent Clostridium difficile infection. Clin Microbiol Infect. 2015;21(3):e23-4. pmid: 25658573 doi: 10.1016/j.cmi.2014.10.005.

449. Porter RJ, Fogg C. Faecal microbiota transplantation for Clostridium difficile infection in the United Kingdom. Clin Microbiol Infect. 2015;21(6):578-82.

450. Quraishi MN, McCune VL, Iqbal T, Pathmakanthan S, Struthers JK, Moran E, et al. Faecal microbiota transplantation via nasogastric route for the treatment of recurrent and antibiotic refractory clostridum difficile infection: The UK experience. Gastroenterology. 2015;148(4):S641-S2.

451. Rao K, Young VB. Fecal microbiota transplantation for the management of Clostridium difficile infection. Infect Dis Clin North Am. 2015;29(1):109-22. pmid: 25677705 doi: 10.1016/j.idc.2014.11.009.

452. Ratner M. Microbial cocktails join fecal transplants in IBD treatment trials. Nat Biotechnol. 2015;33(8):787-8. pmid: 26252119 doi: 10.1038/nbt0815-787.

453. Rossen NG, MacDonald JK, de Vries EM, D'Haens GR, de Vos WM, Zoetendal EG, et al. Fecal microbiota transplantation as novel therapy in gastroenterology: A systematic review. World J Gastroenterol. 2015;21(17):5359-71. pmid: 25954111 doi: 10.3748/wjg.v21.i17.5359.

454. Rupnik M. Toward a true bacteriotherapy for Clostridium difficile infection. N Engl J Med. 2015;372(16):1566-8. pmid: 25875262 doi: 10.1056/NEJMcibr1500270.

455. Russell GH. Too early to determine whether fecal microbiota transplant has therapeutic promise for ulcerative colitis? J Pediatr Gastroenterol Nutr. 2015;60(1):3.

456. Satokari R, Mattila E, Kainulainen V, Arkkila PE. Editorial: a simple faecal preparation for faecal microbiota transplantation--authors' reply. Aliment Pharmacol Ther. 2015;41(3):321. pmid: 25945590 doi: 10.1111/apt.13045.

457. Scaldaferri F, Pecere S, Bruno G, Ianiro G, Laterza L, Gerardi V, et al. An open-label, pilot study to assess feasibility and safety of fecal microbiota transplantation in patients with mild-moderate ulcerative colitis: Preliminary results. Gastroenterology. 2015;148(4):S870.

458. Smith MB, Kassam Z, Burgess J, Perrotta AR, Burns LJ, Mendolia GM, et al. The international public stool bank: A scalable model for standardized screening and processing of donor stool for fecal microbiota transplantation. Gastroenterology. 2015;148(4):S211.

459. Stein D, Rizvi S, Modiri AN, Fang T, Naik AS. Two case reports of toxic megacolon from clostridium difficile infection successfully treated with fecal microbiota therapy. Gastroenterology. 2015;148(4):S645.

460. Stollman N, Smith M, Giovanelli A, Mendolia G, Burns L, Didyk E, et al. Frozen encapsulated stool in recurrent clostridium difficile: Exploring the role of pills in the treatment hierarchy of fecal microbiota transplant nonresponders. Am J Gastroenterol. 2015;110(4):600-1.

461. Surawicz CM. Fecal microbiota transplantation: What we know and what we need to know. Ann Intern Med. 2015;162(9):662-3.

462. Suskind DL. Reply to can fecal microbial transplant effectively treat Crohn's disease? Inflamm Bowel Dis. 2015;21(6):E8.

463. Tauxe WM, Dhere T, Ward A, Racsa LD, Varkey JB, Kraft CS. Fecal microbiota transplant protocol for clostridium difficile infection. Lab Med. 2015;46(1):e19-23. pmid: 25805532 doi: 10.1309/lmci95m0twpdzkod.

464. Tian H, Ding C, Gong J, Wei Y, McFarland LV, Li N. Freeze-dried, capsulized fecal microbiota transplantation for relapsing clostridium difficile infection. J Clin Gastroenterol. 2015;49(6):537-8.

465. Ticinesi A, Turroni F, Nouvenne A, Mancabelli L, Milani C, Morelli I, et al. Clostridium difficile infection and composition of gut microbiota in hospitalized elderly: Case-control study. European Geriatric Medicine. 2015;6:S17-S8.

466. Trubiano JA, George A, Barnett J, Siwan M, Heriot A, Prince HM, et al. A different kind of "allogeneic transplant": Successful fecal microbiota transplant for recurrent and refractory Clostridium difficile infection in a patient with relapsed aggressive B-cell lymphoma. Leukemia and Lymphoma. 2015;56(2):512-4.

467. Tvede M, Tinggaard M, Helms M. Rectal bacteriotherapy for recurrent Clostridium difficile-associated diarrhoea: Results from a case series of 55 patients in Denmark 2000-2012. Clin Microbiol Infect. 2015;21(1):48-53.

468. Vandenplas Y, Pierard D, De Greef E. Fecal Microbiota Transplantation: Just a Fancy Trend? J Pediatr Gastroenterol Nutr. 2015;61(1):4-7. pmid: 25905546 doi: 10.1097/mpg.0000000000000816.

469. Varier RU, Biltaji E, Smith KJ, Roberts MS, Kyle Jensen M, LaFleur J, et al. Cost-effectiveness analysis of fecal microbiota transplantation for recurrent Clostridium difficile infection. Infect Control Hosp Epidemiol. 2015;36(4):438-44. pmid: 25782899 doi: 10.1017/ice.2014.80.

470. Vigvari S, Nemes Z, Vincze A, Solt J, Sipos D, Feiszt Z, et al. Faecal microbiota transplantation in Clostridium difficile infections. Infect Dis (Lond). 2015;47(2):114-6. pmid: 25623242 doi: 10.3109/00365548.2014.969305.

471. Vincent Y, Manji A, Gregory-Miller K, Lee C. A review of management of Clostridium difficile infection: Primary and recurrence. Antibiotics. 2015;4(4):411-23.

472. Vindigni SM, Surawicz CM. The gut microbiome: a clinically significant player in transplantation? Expert Rev Clin Immunol. 2015;11(7):781-3. pmid: 26065325 doi: 10.1586/1744666x.2015.1043894.

473. Vyas D, Aekka A, Vyas A. Fecal transplant policy and legislation. World J Gastroenterol. 2015;21(1):6-11. pmid: 25574076 doi: 10.3748/wjg.v21.i1.6.

474. Wang XJ, Kraft CS, Dhere T. Use of standard donors in fecal microbiotal transplants. Southern Medical Journal. 2015;108(1):68-9.

475. Wei Y, Gong J, Zhu W, Guo D, Gu L, Li N, et al. Fecal microbiota transplantation restores dysbiosis in patients with methicillin resistant Staphylococcus aureus enterocolitis. BMC Infect Dis. 2015;15:265. pmid: 26159166 doi: 10.1186/s12879-015-0973-1.

476. Wei Y, Zhu W, Gong J, Guo D, Gu L, Li N, et al. Fecal microbiota transplantation improves the quality of life in patients with inflammatory bowel disease. Gastroenterol Res Pract. 2015;2015.

477. Weingarden A, González A, Vázquez-Baeza Y, Weiss S, Humphry G, Berg-Lyons D, et al. Dynamic changes in short- and long-term bacterial composition following fecal microbiota transplantation for recurrent Clostridium difficile infection. Microbiome. 2015;3(1).

478. Xu MQ, Cao HL, Wang WQ, Wang S, Cao XC, Yan F, et al. Fecal microbiota transplantation broadening its application beyond intestinal disorders. World J Gastroenterol. 2015;21(1):102-11. pmid: 25574083 doi: 10.3748/wjg.v21.i1.102.

479. Youngster I, Hohmann EL. Fecal microbiota transplantation for Clostridium difficile infection--reply. Jama. 2015;313(7):726. pmid: 25688788 doi: 10.1001/jama.2014.18619.

480. Zainah H, Hassan M, Shiekh-Sroujieh L, Hassan S, Alangaden G, Ramesh M. Intestinal microbiota transplantation, a simple and effective treatment for severe and refractory Clostridium difficile infection. Dig Dis Sci. 2015;60(1):181-5. pmid: 25052150 doi: 10.1007/s10620-014-3296-y.

481. Zhang F, Cui B, Li P, Xu L, Peng Z, Ji G. Scheduled sequential therapy based on fecal microbiota transplantation in steroid-dependent ulcerative colitis: A pilot trial study. Gastroenterology. 2015;148(4):S262.

482. Zowall H, Brewer C, Deutsch A. Projected cost savings of introducing fecal microbiota transplant treatment for clostridium difficile infection in Canada. Value in Health. 2015;18(3):A238.

483. Almeida R, Gerbaba T, Petrof EO. Recurrent Clostridium difficile infection and the microbiome. J Gastroenterol. 2016;51(1):1-10.

484. Hibbeler B. Clostridium difficile: Fecal bacteriotherapy as an option. Dtsch Arztebl Int. 2016;113(5):A185.
