# Supplementary material for: Systematic Review: Adverse Events of Fecal Microbiota Transplantation
Source: PLoS One. 2016 Aug 16;11(8):e0161174. doi: 10.1371/journal.pone.0161174 (PMC4986962; doi:10.1371/journal.pone.0161174)
Supplement: S3 Appendix — (DOC) [file pone.0161174.s003.doc]

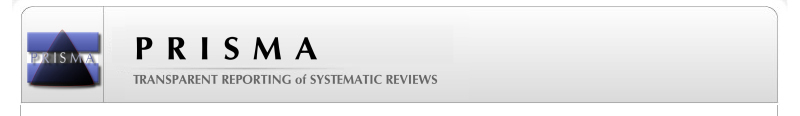
**PRISMA 2009 Flow Diagram**

**Screening**

**Included**

**Eligibility**

**Identification**

Records identified through database searching
(n = 7940)

Additional records identified through other sources
(n = 0)

Records after duplicates removed
(n = 7562)

Records screened
(n = 7562)

Records excluded
(n = 7028)

Full-text articles assessed for eligibility
(n = 534)

Full-text articles excluded, with reasons
(n = 484)

Did not report original clinical data (314)

When the articles did not reported adverse events (166)

When multiple publications reported on the same patients, the most recent and complete data were included (4)

Studies included in quantitative synthesis (meta-analysis)
(n = 50)
